# Design of a nanostructured mucoadhesive system containing curcumin for buccal application: from physicochemical to biological aspects

**DOI:** 10.3762/bjnano.10.222

**Published:** 2019-11-25

**Authors:** Sabrina Barbosa de Souza Ferreira, Gustavo Braga, Évelin Lemos Oliveira, Jéssica Bassi da Silva, Hélen Cássia Rosseto, Lidiane Vizioli de Castro Hoshino, Mauro Luciano Baesso, Wilker Caetano, Craig Murdoch, Helen Elizabeth Colley, Marcos Luciano Bruschi

**Affiliations:** 1 Laboratory of Research and Development of Drug Delivery Systems, Postgraduate Program in Pharmaceutical Sciences, Department of Pharmacy, State University of Maringa, Maringa, Brazil; 2 Department of Chemistry, State University of Maringa, Maringa, Brazil; 3 Department of Physics, State University of Maringa, Maringa, Brazil; 4 The School of Clinical Dentistry, The University of Sheffield, Sheffield, UK

**Keywords:** curcumin, mucoadhesion, oral squamous cell carcinoma, permeation, poloxamer 407

## Abstract

Mucoadhesive nanostructured systems comprising poloxamer 407 and Carbopol 974P^®^ have already demonstrated good mucoadhesion, as well as improved mechanical and rheological properties. Curcumin displays excellent biological activity, mainly in oral squamous cancer; however, its physicochemical characteristics hinder its application. Therefore, the aim of this study was to develop nanostructured formulations containing curcumin for oral cancer therapy. The photophysical interactions between curcumin and the formulations were elucidated by incorporation kinetics and location studies. They revealed that the drug was quickly incorporated and located in the hydrophobic portion of nanometer-sized polymeric micelles. Moreover, the systems displayed plastic behavior with rheopexy characteristics at 37 °C, viscoelastic properties and a gelation temperature of 36 °C, which ensures increased retention after application in the oral cavity. The mucoadhesion results confirmed the previous findings with the nanostructured systems showing a residence time of 20 min in porcine oral mucosa under flow system conditions. Curcumin was released after 8 h and could permeate through the porcine oral mucosa. Cytotoxicity testing revealed that the formulations were selective to cancer cells over healthy cells. Therefore, these systems could improve the physicochemical characteristics of curcumin by providing improved release and permeation, while selectivity targeting cancer cells.

## Introduction

The development of nanostructured systems containing poloxamer 407 (P407) and Carbopol 974P^®^ (C974P) have previously been shown to have rheological and mechanical characteristics beneficial for pharmaceutical and biomedical use [1]. P407 is a non-ionic block copolymer with polypropylene oxide (PPO) and polyethylene oxide (PEO) segments, which can display thermoresponsive properties forming nanometer-sized micelles, hydrogels and lyotropic liquid crystals [2–3]. The increase of temperature can promote the self-assembly from unimers to micelles, with large endothermic heat. In this sense, PPO-groups dehydrate in a hydrophobic core with a surrounding hydrated shell. Higher concentrations of P407 are used (15% to 20%, w/w) as colloidal gelling systems in a cubic hexagonal core with ordered structure, which enable the solubilization of hydrophilic and hydrophobic drugs [4–9]. Moreover, C974P is an acrylic-acid derivative, highly cross-linked, hydrophilic, and displays mucoadhesive properties [1]. In this sense, an increase in temperature promotes the nanometer-sized assembly into three-dimensional micelles with a hydrophobic core of PPO and a hydrophilic shell of PEO that can interact by hydrogen bonds with the hydrophilic acrylic-acid derivative polymer. This results in a binary polymeric system with good viscoelasticity, mucoadhesion, softness and flow properties [1,10–11]. Therefore, different combinations and the characterization of their different functionalities have been reported in the literature [1–3]. These nanostructured systems with mucoadhesive and thermoresponsive properties could provide new properties, including biocompatibility, improved mechanical characteristics tailored for the specific application, in addition to new release mechanisms and improved permeability [9,12–14].

The delivery through the mucosa via buccal administration has shown several advantages as a drug delivery target site. The ease of accessibility for administration and removal, more permeable than skin and containing a rich blood flow and avoidance of first-pass effects, makes this route useful for systemic or local applications [15]. However, the dynamic physiological properties of the oral cavity, such as the variable salivary flow due to different types of stimulation, during mastication, speech and swallowing, could hinder the development of drug delivery systems for this route [15–16]. In order to avoid these drawbacks, nanostructured systems with mucoadhesive polymers, such as acrylic-acid derivatives, have been investigated due to some important characteristics. They can provide intimate contact between the dosage form and tissue, which could guarantee high drug flux through the absorbing tissue to subsequently increase drug permeation and bioavailability [14–15]. In addition, the incorporation of thermoresponsive materials, like P407, could facilitate the administration and preparation of these formulations [10,17].

Head and neck cancer is the sixth most common malignancy worldwide and the prognosis is poor, with a five-year survival of less than 54% and accounting for around 300,000 deaths and more than 550,000 new cases each year worldwide [18–19]. Oral squamous cell carcinoma (OSCC), the primary cause of this cancer type, has been associated with many risk factors, mainly related to excessive intake of alcoholic beverages, tobacco use, high exposure to UV radiation, immunosuppression and age. These risk factors are involved in the transformation of healthy oral mucosal cells (called keratinocytes) to premalignant dysplastic lesions, which can develop to OSCC. Although these lesions can develop in different regions of the oral cavity, including the lip, buccal mucosa, and hard and soft palate, they are most commonly found on the tongue and floor of the mouth [18,20–24]. Despite the advances in conventional methods (chemotherapy, radiotherapy and surgery), the prognosis of OSCC has not improved. In addition, many adverse effects and complications have been reported with current treatment strategies including, facial disfigurement, loss of speech, mastication and swallowing and even in the mildest cases oral mucositis and candidiasis, which drastically reduce the life quality of the patient [21,25]. Early diagnosis by a dentist or physician ensures early treatment to avoid metastatic spread [26–28].

Curcuminoids, derived from curcuma root (*Curcuma longa*), have been used for many centuries in Asian countries as a spice and coloring agent, but also as a medicine [29]. This group of yellow polyphenols are composed of curcumin (CUR), demetoxycurcumin and bis-demetoxycurcumin, which represent 77%, 17% and 3% of the content of the dried extract from curcuma root, respectively [30–31]. CUR has shown anti-inflammatory, antirheumatic and antioxidant activities and it has been used in hepatic and other chronic diseases including diabetes [32]. Recently, the activity of CUR as an anticancer drug has been evidenced and shown to act on a variety of molecular targets that regulate the proliferation and apoptosis, decrease the expression of NF-κB and increase insulin-like growth factor-binding protein 5 (IGFBP-5) and cytochrome P450, family 1, member A1 (CYP1A1) [32–34]. Moreover, CUR can be useful as adjuvant in cancer treatment, after surgical procedure, or in combination with chemotherapy [35–41]. Despite its broad therapeutic potential, CUR has limited stability to light and pH, as well as poor solubility. CUR is also susceptible to degradation due to the formation of phenylate anions and high production of CUR radicals, since this polyphenol has demonstrated high lipophilicity (log P 3.29) and low solubility in aqueous solutions and under alkaline conditions (pH > 7) [30–31,42]. These physicochemical characteristics hinder the bioavailability and therapeutic efficacy of this drug. Consequently, there is a need to associate CUR with carriers to ensure the delivery to the target site and thereby protect the drug from degradation, increase the solubility and provide controlled release as well as permeation, while maintaining biological activity [31,43–45].

To our knowledge, systems containing P407 and C974P have not been investigated to carry CUR and therefore the aim of this study was to develop nanostructured systems containing P407, C974P and CUR to target OSCC. Once developed, we investigated the behavior of the drug in the formulations for their chemical, rheological, mechanical and mucoadhesive characteristics. We also measured the in vitro drug release profile, permeation and the cytotoxic potential of these systems.

## Results and Discussion

### Interaction studies of curcumin in mucoadhesive nanostructured systems

As CUR is highly hydrophobic, unstable and susceptible to degradation by light and pH [31], the interaction of CUR formulations was studied by photophysical methods. The aim was to understand the interaction between this hydrophobic drug with the hydrophobic core of the three-dimensional structure of P407-micelles.

#### Investigation of CUR interaction with the system

**Figure 1:**
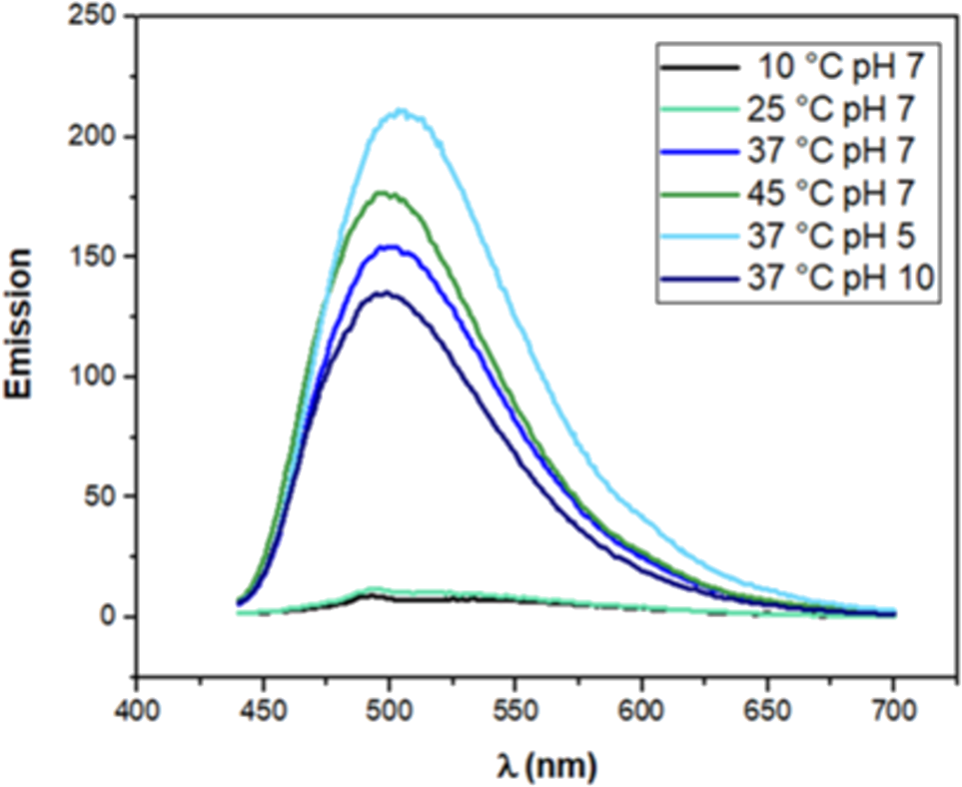
Emission spectra of systems containing 0.01% (w/w) P407, 1.6 × 10^−4^% (w/w) C974P and 1.8 × 10^−5^ mol/L CUR at 10 °C, 25 °C, 37 °C, 45 °C in pH 7, 37 °C in pH 5 and pH 10, obtained with an emission slit of 5–10 nm.

In order to improve the understanding of these interactions between CUR and the formulations, stability assays using photophysical studies were performed to provide information about the interactions of CUR with the polymer blends under different conditions, including the cool storage temperature in refrigerator (10 °C), administration temperature of the formulation to the patient (25 °C), body temperature (37 °C) and over body temperature (45 °C) at pH 7, but also at pH 5 and 10 at 37 °C [46]. The formulations composed of P407, C974P and CUR showed pH 5 during the preparation and before the pH adjustment step, due to the acidic properties of the mucoadhesive polymers [47]. Furthermore, the stability by photophysical studies of polymer blends containing 0.01% (w/w) P407, 1.6 × 10^−4^% (w/w) C974P and 1.8 × 10^−5^ mol/L CUR were spectroscopically evaluated using an emission slit of 5–10 nm. The spectra are displayed in Figure 1. The systems showed a higher emission intensity and larger widths at pH 7, due to the thermal energy employed for CUR encapsulation during the rotary evaporation.

Moreover, the emission intensity of the formulations was lower at pH 10 in comparison to pH 7 at 37 °C. These results could be explained by new chemical species from pKa that do not appear in the emission spectra due to the excitation wavelength of 422 nm (CUR maximum absorption).

Anisotropy of fluorescence (*r*) was also investigated for the samples with maximum peak spectral emission (Table 1).

**Table 1:** Anisotropy (*r*) of formulation containing poloxamer 407 (P407), Carbopol 974P(C974P) and curcumin (CUR) under different conditions of pH and temperature.

Formulation	*r*			
	37 °C (pH 5)	37 °C (pH 7)	45 °C (pH 7)	37 °C (pH 10)

P407/C974P/ CUR	0.2104	0.2517	0.2881	0.2863

Anisotropy is considered a powerful technique to investigate the molecular dynamics of fluorescent solutes, such as CUR. During the fluorescence analysis, the molecules are excited by linearly polarized light, and they absorb and emit the fluorescence in a polarized way. If the emission is highly polarized (approximately or equal to one), the molecules will not rotate between absorption and emission, which would suggest that they are associated with macromolecules. Therefore, the emission of polarization is described as anisotropy [48–49]. Furthermore, this measurement can be used to characterize specific or nonspecific linking as it is dependent on the fraction of fluorescent solute interacting with macromolecules or on the rigidity of the formed complex [48]. Anisotropy values close to zero are related to depolarized emission and intense molecule rotation [49].

Furthermore, the temperature changes were more remarkable than the variation in pH, with the systems investigated at 45 °C displaying higher anisotropy values in comparison to those evaluated at 37 °C.

#### Incorporation kinetics

**Figure 2:**
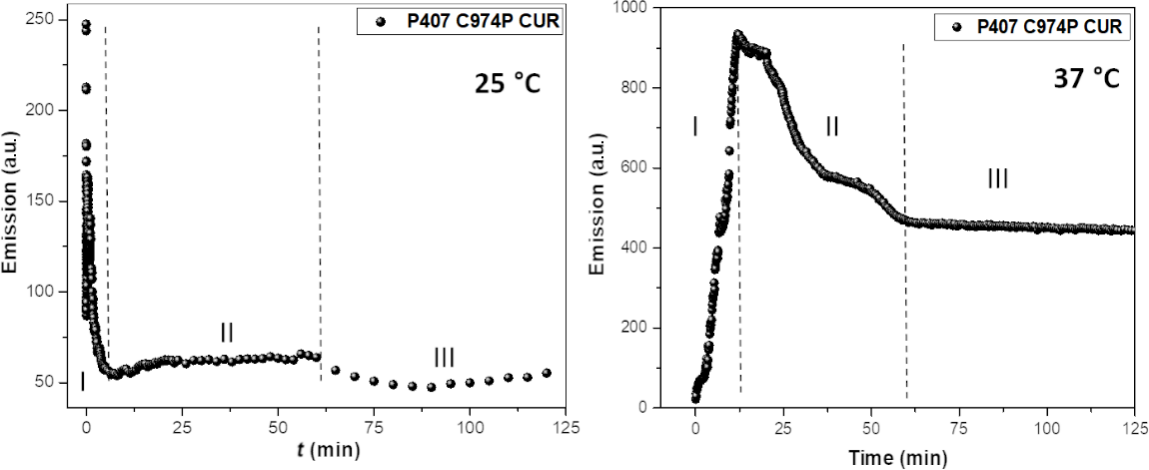
Incorporation kinetic profiles of systems containing poloxamer 407 (P407), Carbopol 974P (C974P) and curcumin (CUR) obtained at 25 °C and 37 °C.

Incorporation kinetics were evaluated by observing the fluorescence emission properties of CUR in order to evaluate if the spectra obtained from the thermal analysis were influenced by the end time of the sample preparation. The time required for CUR incorporation into the polymeric micelles was also determined. Moreover, this analysis allows for the understanding of diffusion and interaction of CUR at a micellar interface. The analyses were carried out in a fluorescence spectrometer with emission and excitation slits of 5–10 nm at 25 °C, once the polymeric micelles were formed, to ensure higher specificity of the results [4]. This evaluation simulates the incorporation of CUR into polymeric systems by the second method of preparation, since the binary polymeric system was prepared and then CUR was incorporated, without pH adjustment at pH 5. On the other hand, by the first method of preparation, the incorporation of CUR was performed during the evaporation of ethanol in the rotary evaporator. Therefore, the incorporation kinetic profile was carried out at 25 and 37 °C, for simulation of the temperature of CUR incorporation by the second and the first preparation method, respectively. Furthermore, the analysis at 37 °C results in the condition where P407 micelles are well-structured. The profiles and kinetic adjustments of CUR incorporation into the polymeric systems is shown in Figure 2.

In general, the effect of temperature did not change the pattern of CUR incorporation. The emission intensity displayed an initial peak with further decrease and maintained emission until the end of the analysis, which hampered the generation of a kinetic pattern for first or second order in both analyzed temperatures. These mechanisms would improve the understanding of the involved mechanisms of this interaction. Kinetic studies of CUR and P407 were carried out by Braga [50], and the initial peak was not observed. Thus, the existence of this initial peak (pattern I) is suggested to occur due to the reorganization of the nanostructured system when CUR is quickly incorporated, as well as the presence of C974P in the micelle structure and subsequent redistribution of CUR in the micellar interface in order to ensure the same concentration of the molecule in the polymeric micelles. Moreover, CUR is incorporated from a highly concentrated medium (ethanolic stock solution) to a low concentrated medium (binary polymeric systems), where a partitioning process take place in a dynamic exchange between CUR molecules incorporated in the micelle and those dissolved in the ethanol until the equilibrium is achieved with decrease in the emission intensity [51]. Two distinct stages (pattern II and III) after pattern I could be observed due to the stabilization of these micellar systems, where CUR becomes aggregated with lower emission intensity.

The temperature was found to influence the velocity of CUR incorporation. This micellar system at 25 °C showed fast incorporation in different locations, at around 1 min, due to the facilitated accessibility of CUR in the monomeric aggregates of P407 and C974P. In addition, lower intensity emission peaks could be observed for these systems at 25 °C, since at lower temperatures PPO groups are hydrated and display weak hydrophobic interactions [6]. On the other hand, the systems evaluated at 37 °C demonstrated slower incorporation, around 60 min, due to the structuring of the micelles to promote a steric hindrance as well as higher emission intensity that promotes increased hydrophobicity of PPO blocks with higher affinity with CUR.

#### CUR localization by fluorescence quenching

**Figure 3:**
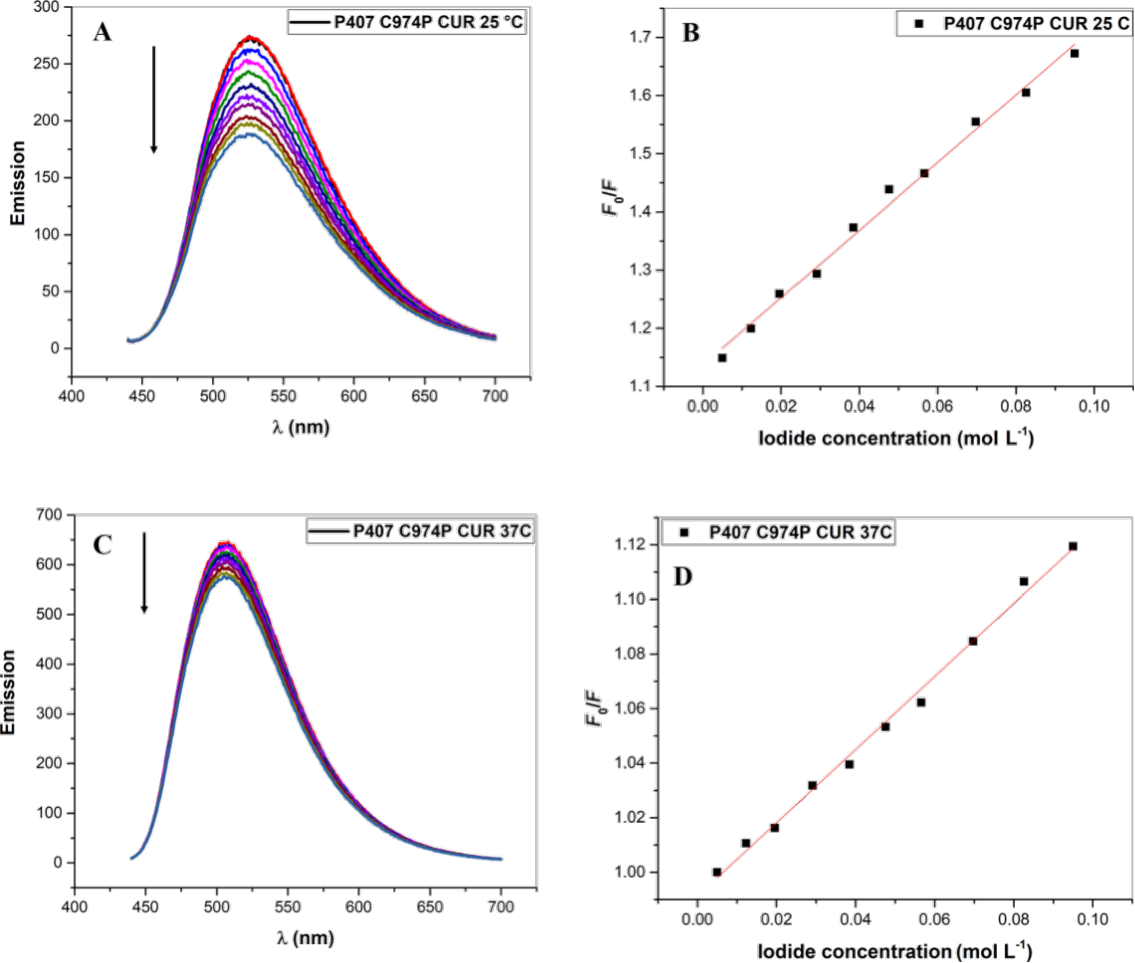
Fluorescence emission spectra and *K*_sv_ determination by Equation 1 of CUR (3.6 × 10^−5^ mol/L) at 25 °C (A and B) and 37 °C (C and D) for P407 (0.2%, w/w) and C974P (3.2 × 10^−4^%, w/w); λ_exc_ = 422 nm with a 5–10 nm slit width. The spectral variation of CUR with I^−^ concentration as determined by fluorescence emission at 25 °C (B) and 37 °C (D). The arrows in (A) and (C) indicate the fluorescence suppression direction of CUR with I^−^ concentration.

The localization of CUR in the polymeric systems was performed by fluorescence quenching, using iodide as a hydro-soluble suppressor [2,52–54]. The studies were performed at 25 °C (room and administration temperature) and 37 °C (body temperature). Using this assay, it is possible to evaluate the location of CUR in the polymeric system during drug release and to determine the location of encapsulated drugs. If the drug is located in an external location, it forms a complex with iodide, and consequently, the intensity of the spectra decreases [52]. The fluorescence quenching studies were carried out with formulations composed of 0.02% (w/w) P407, 3.2 × 10^−4^% (w/w) C974P and 3.6 × 10^−5^ mol/L CUR prepared by solid dispersion and stored at 25 °C (Figure 3). Considering the high concentration of water, this analysis was not influenced by the viscosity of the formulations. Moreover, the system was constantly agitated with a magnetic stir bar.

In the spectra shown in Figure 3, it was evident that formulations evaluated at 37 °C showed a higher spectral intensity in comparison to studies performed at 25 °C. These results can be explained by the structure of thermoresponsive systems in response to an increase in temperature. Moreover, for the formulations evaluated at 25 °C, a decrease in emission was observed during iodide addition, which demonstrates the higher accessibility of the hydro-soluble suppressor, and consequently, higher collisions with CUR [52,54–55]. However, the spectra of the systems evaluated at 37 °C exhibited overlapping peaks, which demonstrates that CUR is not accessible to complex with the hydro-soluble suppressor. This suggests that the CUR is localized in the micelle core at 37 °C, which results in a slower release from the nanostructured system.

High Stern–Volmer constant (*K*_sv_) values are correlated with the CUR being externally located, with accessibility for complexation with iodide. Conversely, low *K*_sv_ values suggest an internal location, and consequently, no accessibility to the suppressor molecule. The system evaluated at 37 °C displayed lower *K*_sv_ values (*K*_sv_ = 1.3403 mol/L), whereas formulations evaluated at 25 °C showed higher *K*_sv_ values (*K*_sv_ = 5.8090 mol/L).

In this context, systems containing C974P displayed spectroscopic characteristics that favored further studies, as they presented lower *K*_sv_ values, indicating internal location of CUR in P407-micelles as well as higher anisotropy values. This result suggested that the viscosity of the microenvironment helped to obtain stronger interactions. In addition, the system stored at 25 °C also showed interesting results, in that they did not show any visible sign of CUR precipitates after 15 days of storage (Figure S1 in Supporting Information File 1), indicating that CUR was in an internal location.

#### Morphological analysis by scanning electron microscopy

**Figure 4:**
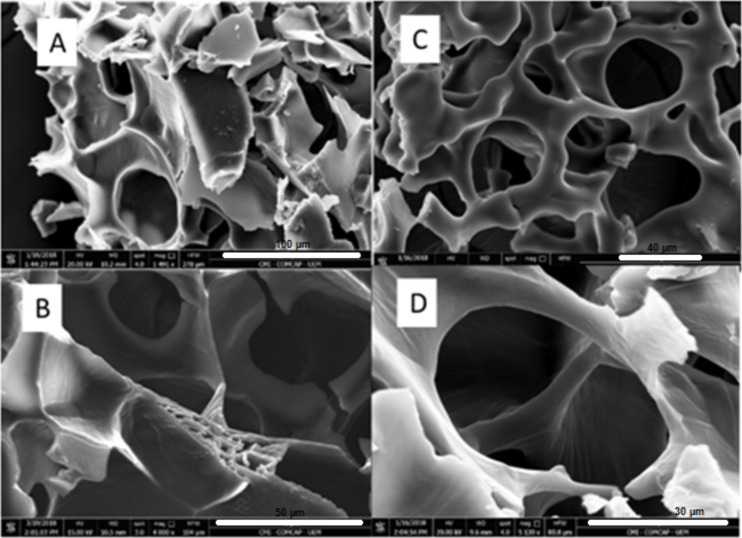
SEM micrographs of systems containing 15% (w/w) P407, 0.25% (w/w) C974P: without CUR original magnification ×1500 (A, scale bar = 100 µm) and ×4000 (B, scale bar = 40 µm); containing CUR original magnification ×1800 (C, scale bar = 50 µm) and ×5000 (D, scale bar = 30 µm).

The morphological characteristics of the preparations with and without CUR were evaluated by scanning electron microscopy (SEM) (Figure 4). Micrographs of formulations containing P407 and C974P revealed polymeric fragments with heterogeneous, but well defined, structures. This was probably due to the presence and movement of water that was removed due to the freeze-drying process. Besides, the portion of the microstructures or microchannels exhibiting exposed breakage (Figure 4B) were probably due to the interaction between P407 and C974P.

SEM micrographs of preparation containing CUR showed the presence of more irregular structures and channels without defined orientation. These data can be explained by the preparation method since the samples were frozen at −20 °C, where restructuring of the polymer can be observed. Moreover, the negative charge of C974P and CUR hindered the exploration of the structure at higher magnification due to the interaction of these components and the microscopic filaments.

#### Morphological analysis by transmission electron microscopy

**Figure 5:**
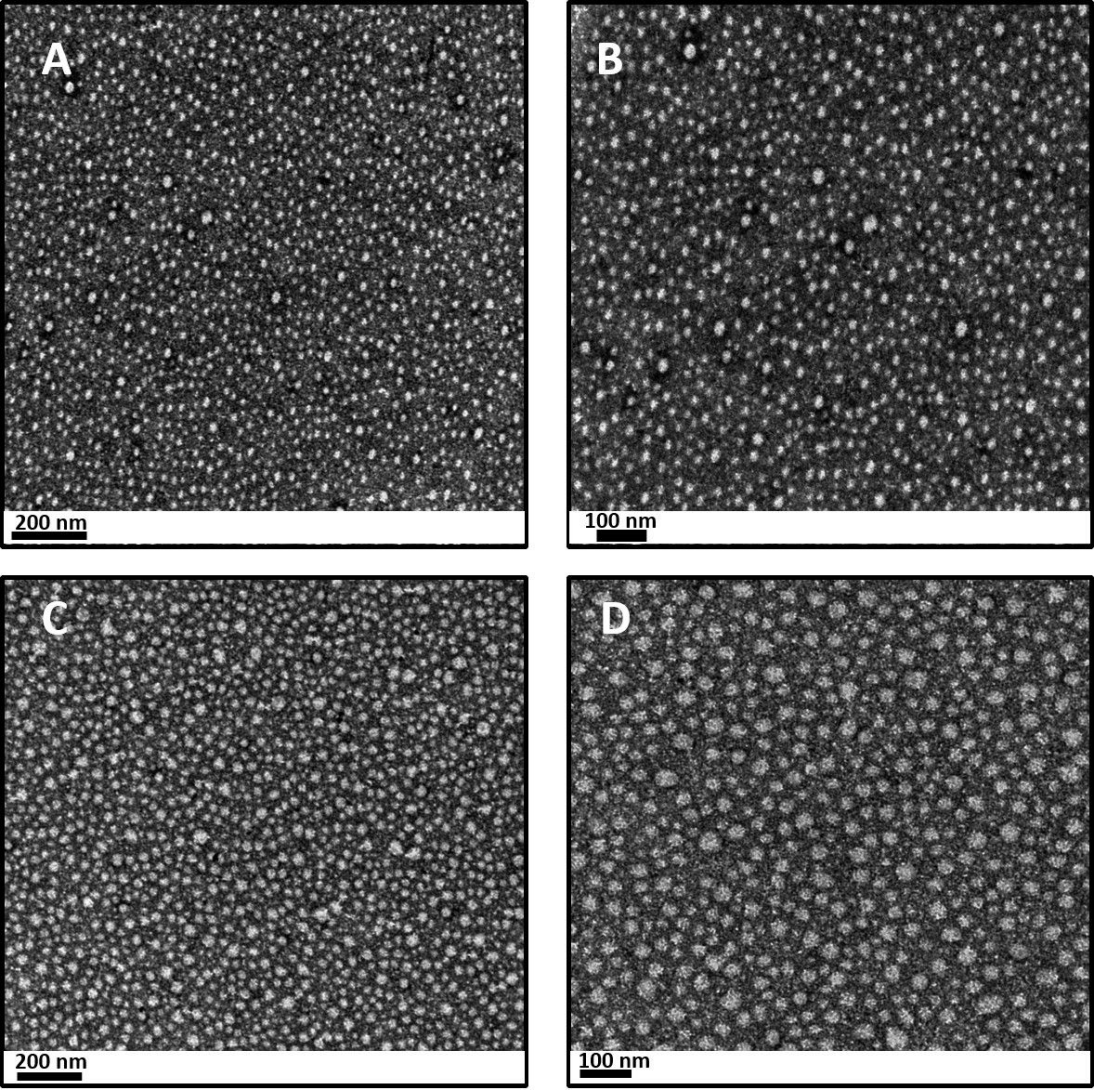
TEM images of nanostructured systems at 37 °C demonstrating the individual micellar organization: (A) and (B) represent the nanostructured systems in the absence of CUR, scale bar equals to 200 and 100 nm, respectively. (C) and (D) represent the nanostructured systems containing CUR, scale bar equals to 200 and 100 nm, respectively.

The nanostructured organization of the polymeric systems is evidenced in Figure 5. In the absence of CUR, the micelles (Figure 5A and 5B) are represented by the white spherical shapes of approximately 20 nm in diameter. Some authors have performed transmission electron microscopy (TEM) of P407-systems and showed smaller micelles (≈10 nm) with higher homogeneity [56–58].

These differences in the micelle size could be due to the presence of C974P and interactions between PEO segments of P407 and hydroxyls of C974P.

Moreover, the TEM images of the nanostructured systems containing CUR have been obtained at 37 °C (Figure 5C and 5D). Considering that CUR is found in the hydrophobic PPO core, as demonstrated in the localization studies, it was observed to have better homogenization of the size and shape of the micelles in comparison to the systems without the drug. Additionally, the micelles were larger (≈40 nm), probably due to the interaction of PPO and CUR. In this sense, the presence of CUR promoted a better structuring of the system.

#### Micelle size analysis

The micelle size, polydispersity index (PDI) and D90% of the nanostructured systems with and without CUR were determined by dynamic light scattering (DLS) and the results are displayed in Table 2.

**Table 2:** Micelle size (*D*), polydispersion index (PDI) and micelle size distribution (D10%, D50% and D90%) of the nanostructured systems with and without CUR, evaluated at 25 °C and 37 °C^a^.

System dilution (%, w/w)^b^	Analysis	Temperature			
		25 °C		37 °C	
		P407/C974P	P407/C974P/CUR	P407/C974P	P407/C974P/CUR

0.3	*D* (nm)	26.87 ± 5.37	12.83 ± 0.12	14.50 ± 6.32	19.23 ± 0.06
	PDI	1.04 ± 0.04	0.39 ± 0.02	0.24 ± 0.02	0.23 ± 0.01
	D10% (nm)	21.70 ± 0.00	10.77 ± 0.06	10.97 ± 0.31	15.50 ± 0.17
	D50% (nm)	21.87 ± 0.70	11.77 ± 0.06	13.23 ± 0.76	18.17 ± 0.12
	D90% (nm)	25.57 ± 3.39	15.47 ± 0.15	18.20 ± 1.21	22.30 ± 0.10

1.5	*D* (nm)	5.10 ± 0.17	15.90 ± 1.31	13.50 ± 0.35	11.33 ± 0.64
	PDI	0.29 ± 0.01	0.17 ± 0.00	0.14 ± 0.02	0.19 ± 0.01
	D10% (nm)	3.73 ± 0.15	11.53 ± 1.06	9.83 ± 0.21	8.37 ± 0.42
	D50% (nm)	4.57 ± 0.21	14.00 ± 1.28	12.13 ± 0.38	10.20 ± 0.44
	D90% (nm)	6.30 ± 0.26	20.20 ± 1.67	17.03 ± 0.47	14.50 ± 0.53

^a^Results represents the average of at least three replicate analyses. ^b^In relationship to P407 amount in the formulation.

The polymer concentration should be low enough to circumvent multiple scattering in DLS measurements [56]. Thus, the systems were diluted in two different concentrations, 0.3% and 1.5% (w/w), in relationship to the amount of P407 in the formulations. It was observed that the system is quite dynamic. Thus, the most diluted system (0.3%, w/w) showed a significantly higher micelle size and PDI (*p* < 0.05), probably due to the presence of water, which can enable higher hydration of the polymeric chains. The presence of CUR significantly reduced the PDI (*p* < 0.05) for the system diluted at 0.3% (w/w) P407 at both temperatures, and at 37 °C for the system diluted at 1.5% (w/w) P407. Moreover, the presence of CUR did not change the PDI for the different P407 dilutions (0.3 and 1.5%, w/w) at 37 °C. The presence of CUR decreased the micelle size (*D*) in the 0.3% (w/w) P407 dilution at 25 °C, but significantly increased *D* for all other conditions of (*p* < 0.05). Thus, the increase in the P407 concentration results in nanometer-sized micelles with a lower PDI. In addition, the presence of CUR increases the micelle size and also results in a low PDI. The TEM results and particle size when diluted to 0.3% (w/w) P407 were similar (but not the same) due to water removal during the sample preparation for TEM analysis [56]. Therefore, the results showed the nanometer-sized structuring of the systems.

#### Rheology

**Figure 6:**
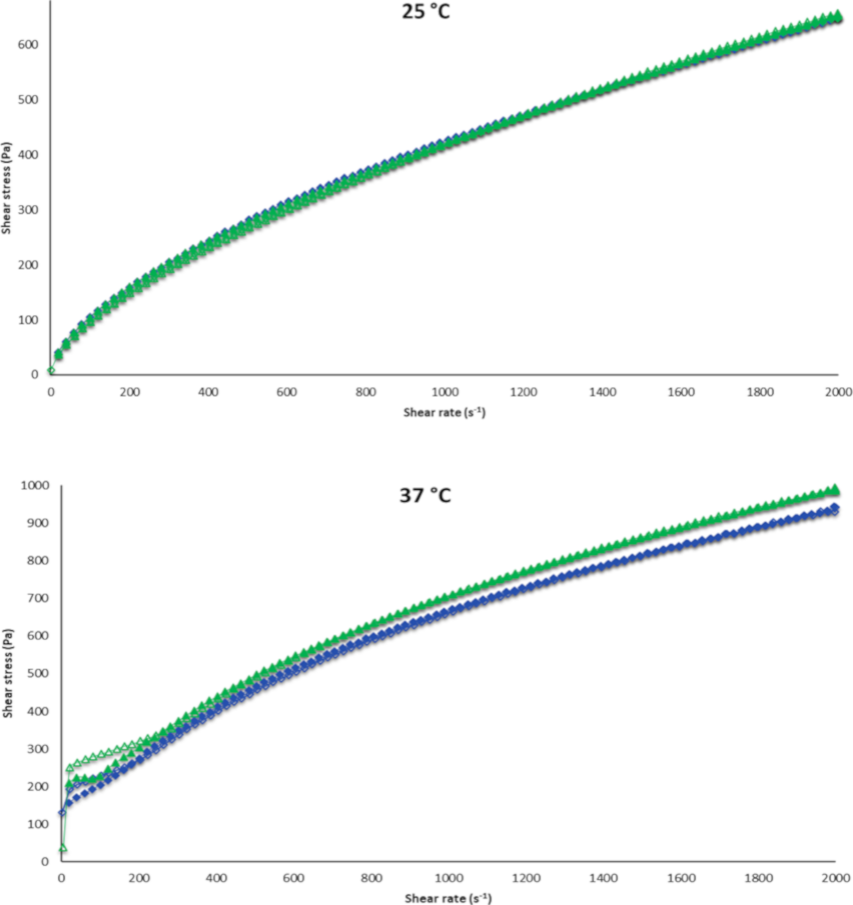
Flow rheograms of formulations containing 15% (w/w) P407, 0.25% (w/w) C974P (∆ - green) and 15% (w/w) P407, 0.25% (w/w) C974P and 0.08% (w/w) CUR (◊ - blue). The closed symbols represent up-curve and open symbols represent down-curve. Error bars have been omitted for clarity, and each rheogram represents the average of at least three replicates with variation coefficient 10%.

The flow properties of binary polymeric systems containing 15% (w/w) P407, 0.25% (w/w) C974P, 0.08% (w/w) CUR were evaluated at 25 and 37 °C (Figure 6).

The nonlinear behavior to shear stress due to the shear rate (non-Newtonian), resulting in structural changes, was maintained even after the incorporation of CUR in the polymer blends. Moreover, the addition of CUR in binary polymeric systems did not lead to a change in flow rheological profiles at 25 °C, whereas a slight decrease of shear stress was observed for systems evaluated at 37 °C. In this way, CUR did not change the structuring of the system. Conversely, the increase in temperature leads to an increase in shear stress due to the thermoresponsive properties of the preparations.

Low hysteresis areas and different yield values could be observed in a prominent way at 37 °C, in comparison to systems investigated at 25 °C. Consequently, these systems showed shear thinning behavior flow, which is considered to be a desirable characteristic for pharmaceutical semi-solid formulations in order to facilitate clinical administration in a uniform way over the mucous tissue of the buccal cavity. Subsequently, it can recover the rheological properties that these systems presented before the shear stress application [10,59–60].

The effect of the presence of CUR and the increase in temperature were statistically evaluated by the consistency index (*K*), flow behavior index (*n*) and yield value. These indexes were calculated by the rheological models, Ostwald de Waele, Casson and Herschel–Bulkley. In order to verify the rheological model that could properly fit the *K* and *n* value, *R* or Χ^2^ were calculated and it was observed that *R* values were closer to 1, according to Herschel–Bulkley theory (Table 3).

**Table 3:** Consistency index (*K*), flow behavior index (*n*), yield value and hysteresis area at 25 and 37 °C for systems containing P407, C974P with and without CUR.

Rheological properties	Temperature (°C)	P407/C974P/CUR (%, w/w)	
		(15/0.25/0)	(15/0.25/0.08)

*K* (Pa·s)*^n^*^ a^	25	5.4027 ± 0.2110	6.1910 ± 0.6374
	37	15.8867 ± 0.7379	15.4367 ± 0.8164
*n* (dimensionless)^a^	25	0.6311 ± 0.0058	0.6094 ± 0.0130
	37	0.5371 ± 0.0030	0.5395 ± 0.0068
Yield value (Pa)^a^	25	7.7960 ± 0.4634	2.2065 ± 0.8565
	37	48.6033 ± 3.0647	26.4000 ± 0.5200
Hysteresis area (Pa/s)^a,b^	25	7036.80 ± 2423.70	14381.16 ± 3607.37
	37	−17520.00 ± 8553.99	−26805.00 ± 2725.00

^a^Each number represents the mean of at least three replicates; ^b^Positive number represents rheopexy and negative numbers represent thixotropy.

The presence of CUR did not lead to any significant differences (*p* = 0.6875) in the consistency index (*K*) of the preparations, and the formulations kept the same resistance to deformation [11], confirming that CUR is located in the core of the polymeric micelle (as shown by the localization analysis) and therefore did not alter the interactions between P407 and C974P in order to change the viscosity. Otherwise, the increase in temperature led to the significant increase of *K* (*p* = 0.000210) due to the thermoresponsive properties of P407 [4].

The formulations with and without CUR showed shear thinning behavior due to *n* values lower than 1 (Table 2). The positive yield values demonstrated that these systems are plastic [9]. Thus, the increase in temperature significantly decreased the *n* values (*p* < 0.05), whereas the presence of CUR had no significant influence on the *n* values (*p* = 0.138912).

The yield value and hysteresis area results are shown in Table 2. The yield value demonstrates the ability of the formulations to withstand a significant shear stress without flow, and then after the weakening of the structure, the ability to start to flow [61]. Both an increase in temperature and incorporation of CUR led to significant differences (*p* < 0.05) in the yield value. The significant increase in yield value with an increase in temperature and subsequent micelle structuring can be explained by the transition from liquid to gel. However, the increase of CUR led to a significant decrease in the yield value, probably due to the higher exposure of the hydrophilic portion to water [62–63].

The hysteresis area of the formulations was investigated by RheoWin 4.10.0000 software (Haake^®^). The formulations displayed thixotropic behavior at 25 °C, which provides resistance to breakage in addition to higher structural flexibility, which is due to the lower viscosity after the application of shear stress [64–65]. Conversely, the formulations exhibited a significant decrease (*p* < 0.05) in the hysteresis area at 37 °C (Tukey). Thus, both formulations (with and without CUR) demonstrated rheopetic behavior due to the higher influence of P407 in the structuring of the system at 37 °C. This behavior is considered important in order to increase the retention of preparations in the buccal cavity [59–60]. Moreover, the presence of CUR significantly decreased the hysteresis area (*p* < 0.05) at 37 °C. These systems displayed rheopexy at 37 °C, which are negative values. In this sense, the decrease in the hysteresis area means that the systems showed higher rheopexy areas. The presence of CUR resulted in an increase in the micelle size and homogeneity, as evidenced by TEM and the size analysis. Thus, an improved organization and higher resistance to the stress and shear rate applied were observed. This higher resistance to the flow explains the higher rheopexy of the systems containing CUR, mainly at 37 °C.

**Figure 7:**
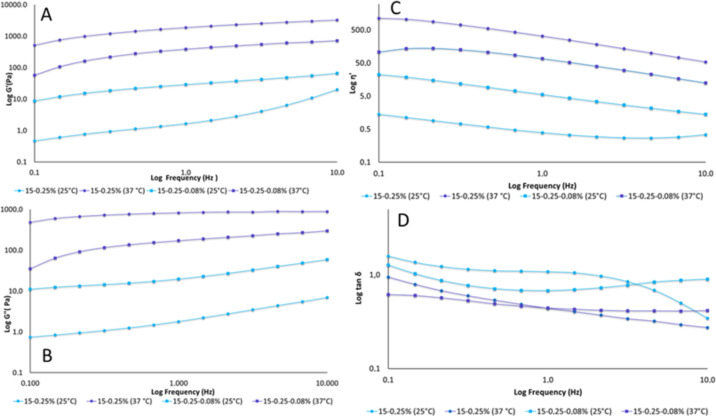
(A) Elastic modulus, G’ (Pa), (B) viscous modulus, G” (Pa), (C) dynamic viscosity, η’ (Pa), and (D) tan δ of the formulations containing P407 (15%, w/w) and C974P (0.25%, w/w) with and without CUR (0.08%, w/w) evaluated at 25 and 37 °C. Each point represents the average of at least three replicates with a variation coefficient lower than 10%.

The effects of the presence of CUR and the change in temperature on the viscoelastic properties (G’, G”, η’ and tan δ) of the systems was also evaluated (Figure 7).

An increase in frequency for both the elastic and viscous modulus was observed (Figure 6A and 6B). A decrease in the dynamic viscosity and loss tangent were observed with increasing frequency (Figure 6C and 6D). The exception is for the loss tangent of the CUR systems evaluated at 25 °C, which remain constant at the majority of the frequencies. Moreover, the increase in temperature led to the increase of G’, G”, η’ and a decrease in tan δ for formulations with and without CUR. This behavior has already been previously observed for systems containing P407 and other acrylic acid derivatives [9,11], as well as polymer blends containing P407 and C974P or PCB [1,10].

Regarding the effect of the presence of CUR at 25 °C, an increase of G’, G” and η’ and a decrease of tan δ was observed for most frequencies. Hence, these changes provided better structuring and elasticity at 25 °C. However, polymer blends containing CUR investigated at 37 °C displayed lower G’, G” and η’ with an absence of significant changes for tan δ. These results could be explained by the difficult micelle structuring of P407 and jellified three-dimensional chains.

Furthermore, both preparations exhibited viscoelastic behavior, except for the polymer blends containing P407 and C974P without CUR at 25 °C. In these formulations, the G’ values exceeded G” and the loss tangent was smaller than 1 for the viscoelastic preparations. Thus, the viscoelasticity is favorable to oscillatory movements performed at 25 °C, occurring during transport and storage of formulations [9].

The gelation temperature, *T*_sol–gel_, of the formulations with and without CUR was investigated as well. The systems displayed lower G’ values at low temperatures; however, high G” values were observed as the temperature was increased. Even with the incorporation of CUR, the dynamic viscosity increased significantly due to the increase in temperature and gelation temperature [59,66]. The presence of CUR significantly increased (*p* < 0.05) the gelation temperature of the preparations from 36.03 ± 0.06 °C in systems without CUR (15/0.25) to 36.94 ± 0.12 °C in systems with CUR (15/0.25/0.08). Consequently, the structuring of the jellified three-dimensional chain is explained by the difficulty of the externalization of the hydrophilic portion (i.e., ethylene oxide (EO)) of micelles to interact with water and initiate the interaction between EO and C974P-hydroxil [43]. Despite the significant increase in the gelation temperature of the formulations containing CUR, the *T*_sol–gel_ is considered suitable (between 25 and 37 °C).

#### Texture profile analysis

The mechanical properties of the preparations with and without CUR were evaluated by texture profile analysis (TPA). The information about the physical structure of gels obtained by TPA are useful for the development of nanostructured mucoadhesive systems related to the preparation, packaging, administration and structuring from development until the application and performance evaluation at the application site. In this way, the formulations should be resistant to the forces applied by the environment. This is also true for saliva, which can also be considered as a natural protection in the organism against impurities exposed to the mucosa and can hinder the retention of such systems and thereby impair the clinical efficacy [65–67].

**Figure 8:**
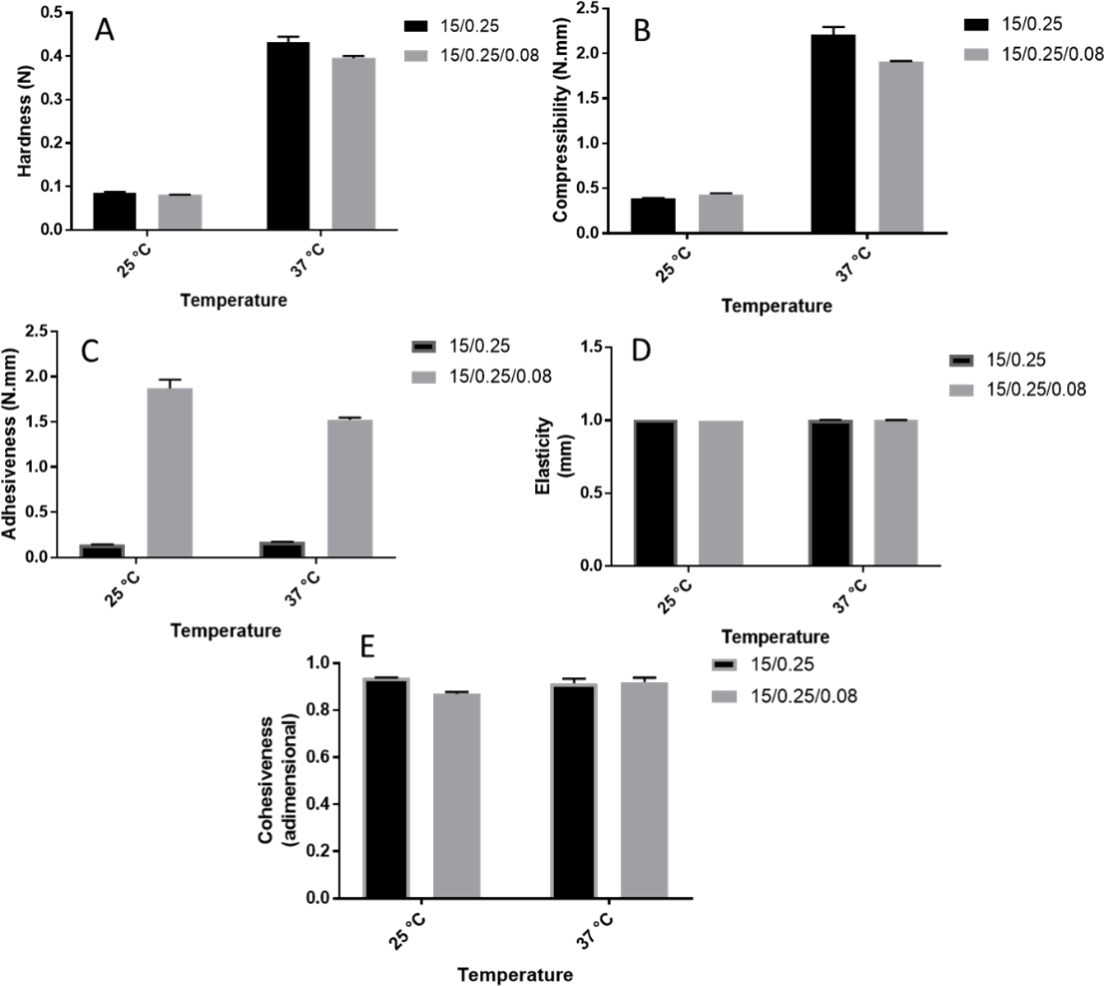
Textural properties of the systems containing P407, C974P and CUR, investigated at 25 and 37 °C: (A) hardness, (B) compressibility, (C) adhesiveness, (D) elasticity and (E) cohesiveness. Each value represents at least three replicates.

Hardness, compressibility, adhesiveness, elasticity and cohesiveness results for the formulations with or without CUR at 25 and 37 °C are given in Figure 8. In addition, the effects of the presence of CUR and the increase in temperature were statistically evaluated for each parameter.

Hardness is an indicator of the ability to remove the formulation from the packaging material and its subsequent spreadability on the mucous tissue in a uniform layer to avoid any discomfort to the patient. Conversely, compressibility expresses the application of the formulation in the buccal cavity. Low compressibility and hardness values are desirable in order to facilitate the removal of the preparation from the packaging material and administration in the buccal cavity and topically applied over the mucosa [66–68]. However, formulations should display enough resistance to avoid the product flowing from the packaging material and application site [10]. Adhesiveness, considered as the required work necessary to overcome the attraction forces between the sample surface and the polycarbonate probe surface, is a desirable parameter for mucoadhesive preparations aimed at buccal applications. In this sense, a higher adhesion could implicate higher retention, and consequently, higher clinical efficacy of preparations for buccal drug [67–68].

The incorporation of CUR significantly decreased (*p* < 0.05) the hardness and compressibility, which is a desirable characteristic to facilitate application of the formulation in the buccal cavity. This behavior has been observed previously for polymer blends containing P407 and other acrylic acid derivative with a similar cross-linking degree as C974P to carry hypericin [69]. In this study, the polymeric micelle relaxation due to the hydrophobic drug could explain this behavior and be related to the low yield values in the flow rheology analysis. On the other hand, CUR did not significantly (*p* = 0.06597) influence the adhesiveness values of different preparations. This phenomenon has been observed for other polymer blends containing hydrophobic drugs [7] since the mucoadhesive properties are obtained by the interaction between the hydrophilic portion of the polymeric micelles and mucosa [70].

It was also observed that an increase in temperature significantly (*p* < 0.05) increased hardness, compressibility and adhesiveness due to the thermoreversilibity of the material, which favors the structuring of the systems, as previously observed in the flow rheology with higher consistency index numbers. These higher values are desirable for a higher adhesion to the buccal cavity, leading to longer contact time. These results confirm that the system is mucoadhesive but also warrant further mucoadhesion investigations [11,59,71].

The gel ability to flow and return to the initial state is defined as elasticity [9]. In addition, cohesiveness is related to the restructuration and molecular interactions after subsequent shear stress during the application of the system [67,72]. The incorporation of CUR did not significantly influence (*p* < 0.05) the elasticity and cohesiveness, which could be explained by the influence by hydrogen bonds and water mobility in the sample. As elucidated in location studies, CUR is a hydrophobic molecule that is located in the core of polymeric micelles and thus it should not influence this parameter [9]. Regarding the temperature effect (at 25 and 37 °C), the preparations demonstrated elasticity and cohesiveness values significantly lower (*p* < 0.05) at 37 °C.

#### Syringeability

The work required to expel the formulation from a syringe (syringeability) at 25 °C was investigated by a texture analyzer. This test was performed in order to simulate extrusion of the formulation from the packaging material and during the administration over a lesion in the buccal cavity [59]. The effect of CUR incorporation in this system was evaluated, and despite the fact that the formulation containing CUR displayed a lower syringeability (32.6383 ± 2.1814 N·mm), it was not significantly lower (*p* > 0.05) than the syringeability of preparations without CUR (34.0390 ± 1.3390 N·mm). Thus, CUR incorporation did not influence the syringeability of the formulations and the results indicated the ability to administer the system [59].

### Mucoadhesive properties

#### In vitro evaluation of mucoadhesive strength by detachment force

The mucoadhesive characteristics of preparations with and without CUR were evaluated by detachment force using a partially hydrated mucin disc as substrate [59,70]. According to this method, it is possible to obtain a graph with the force required to separate two surfaces with time and the maximum required force to separate the formulation from the mucin disc (mucoadhesive force). In addition, the adhesion work values were calculated.

The incorporation of CUR in mucoadhesive thermoresponsive systems led to a significant increase (*p* < 0.05) for both the mucoadhesive force from 0.2109 ± 0.0054 N without CUR to 0.2175 ± 0.0016 N with CUR and increase in the adhesion work from 0.6890 ± 0.0377 N·mm without CUR to 0.7667 ± 0.0475 N·mm with it incorporated. However, these results were not observed for adhesiveness, which could provide evidence of adhesion. This parameter evaluates the interaction between the formulation and polycarbonate probe, where this lower specificity explains this result. Conversely, the detachment force method relates the interaction of C974P-hydroxyls and the mucin chain. This is provided by the nanostructuring of polymer blends at body temperature, and hence, a P407 micelle structure with a hydrophobic nucleus and hydrophilic shell, which interacts with a mucoadhesive polymer in the external portion of polymeric micelles [10]. Moreover, these results are clinically relevant since the formulations containing CUR would display a longer residence time than the systems in the absence of this drug. Hence, the formulation should demonstrate the intimate contact with the oral mucosa, for prolonged periods, which could provide the concentration of the drug close to the cancer lesions with higher bioavailability [73].

The calculation of mucoadhesive force and adhesion work has already been discussed by other authors [70] since mucoadhesive force is the most commonly used parameter to describe mucoadhesion. However, adhesion work seems to be influenced by the elasticity and plasticity of the investigated systems. It is suggested in the literature as the most suitable term for the evaluation of the detachment force since it can better detect the differences in mucoadhesive ability [74], as was observed for the formulations with and without CUR. Moreover, the work calculation (if converted to units of Joule) reflects the necessary energy to separate two surfaces. Thus, the significant increase of adhesion work correlates with higher interaction between the mucin surface and the preparations containing CUR.

#### Ex vivo mucoadhesive properties by falling liquid

**Figure 9:**
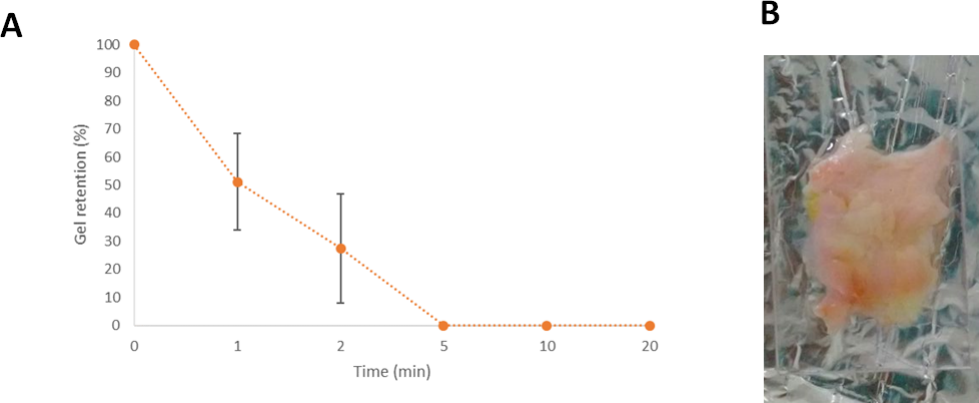
(A) Cumulative retention profile of a polymeric system containing poloxamer 407, Carbopol 974P^®^ and curcumin retained in a mucous layer versus time. (B) Oral porcine mucosa after the end of the test.

Besides the detachment force, the determination of the mucoadhesive properties of nanostructured systems could also be explored by liquid falling experiments [30,74–75]. This method is related to the ability of systems stay adhered to surface mucosa with the falling liquid (PBS buffer) at 4 mL/min during 20 min. Higher flow resistance evidences strong adhesive interactions between preparations and mucosa. The amount of adhered gel is calculated in an indirect method. Thus the formulation eluted with buffer in a beaker containing Tween 80 and the drug in the formulation was quantified by chromatographic methods. Consequently, it was evaluated only for the CUR systems. The retention of the systems without CUR have already been evaluated by a similar method, where the formulations were marked with FITC-dextran and the retention was investigated by fluorescence microscopy [70]. The cumulative formulation percentage adhered versus time is displayed in Figure 9.

The time buffer elution effect as a function of time over the sample was statistically investigated and it was verified to significantly decrease (*p* < 0.05) the gel retention with time. Besides, the high variability observed is due to the irregularities of the mucosa surface. Thus, the formulation has already been eluted completely after 5 min. Even if the methodology used to evaluate the retention of the formulations without CUR was different [70], it is possible to compare the results, since the PBS flow rate used was the same (4 mL/min). In this sense, the tests carried out by Bassi da Silva and collaborators [70] demonstrated that the systems were eluted completely after 5 mL elution, which corresponds to 1 min 15 s. In this sense, the systems containing CUR demonstrated improved retention in comparison with the systems without CUR. This result agrees with the mechanical and rheological characteristics observed. One of the replicate images is showed in Figure 9B.

#### In vitro drug release profile

During the development of drug delivery systems for buccal application, in vitro drug release is highly important and is considered a prerequisite for absorption as it contributes to the rate and extension of drug bioavailability in the body [76]. Moreover, these investigations can distinguish between different systems containing the same drug, the same formulations after aging, and process changes during the preparation process [17,72].

CUR release profiles were obtained by the continuous monitoring of drug release over time. The key factors which could affect CUR release are release media volume, temperature and agitation or flow and cellulose acetate membrane [77]. Thus, in vitro drug release of CUR from the mucoadhesive nanostructured systems was carried out with controlled temperature and agitation in order to simulate conditions in the buccal cavity without regard to other physiological aspects (pH, salt concentration, enzymes and mouth movements during swallowing and speaking). These initial tests are important to verify if the drug could be released from the drug delivery system and how the applied technology influences the availability of the drug.

Another aspect to be considered for the in vitro drug release analysis is the choice of release medium. For hydrophobic drugs, sometimes it is necessary to add surfactants that can provide the sink condition. In this study, Tween 80 was used as a surfactant, as previously used in other in vitro CUR drug release investigations [30,78]. Tween 80 can interfere with the structure and rheological properties of the peripheral area of the gel. However, this situation is similar to in vivo conditions, where other substances with surfactant properties can be present in buccal environment [59,79].

**Figure 10:**
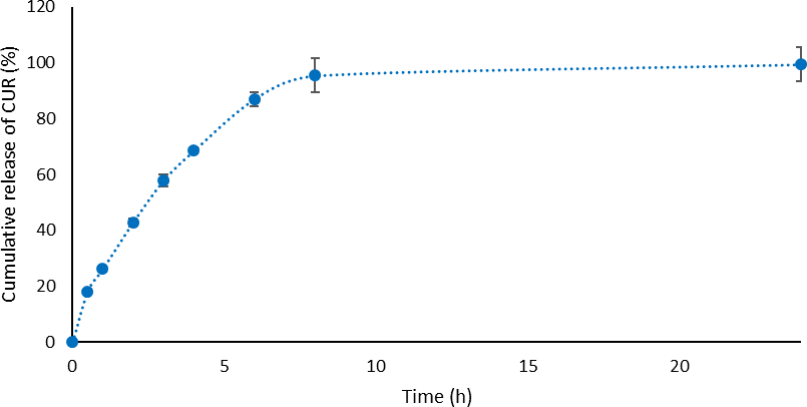
Release profile of CUR from a mucoadhesive nanostructured system containing 15% (w/w) P407, 0.25% (w/w) C974P and 0.08% (w/w) CUR.

The complete release of CUR (100%) occurred after approximately 8 h (Figure 10), making it suitable for buccal applications. The general equation (Equation 2) described for polymeric systems [80] was used to evaluate the release mechanism of CUR. Here, the release exponent (*n*), which determines if the drug release mechanism is Fickian (Case I) or non-Fickian (transport Case II, anomalous or super case II) revealed an *n* value of 0.6517. The nanostructured systems displayed anomalous release kinetics, hence, the polymeric chains were slowly reorganized, whereas CUR diffused by time-dependent anomalous effects. The solvent diffusion velocity displayed similar relaxation of the polymeric chains [77].

#### Ex vivo permeation of curcumin in porcine oral mucosa

Permeation studies are considered fundamental to determine the viability of oral mucosa as a targeting site for drug delivery [81–82]. These studies can be performed ex vivo, in vitro or in vivo and are dependent of the drug physicochemical characteristics and its behavior when incorporated in drug delivery systems and biological target tissue. The buccal cavity presents significant differences in permeability due to the composition and thickening of the mucosa [81–82].

Different animal species display varying thickness and keratinization patterns with porcine oral mucosa the most commonly employed for ex vivo testing due to the physiological similarities with human tissue, ethical considerations and low cost [81–83].

Permeation assay using a Franz cell is a quantitative technique where the amount of drug in the receptor medium is measured according to its physicochemical characteristics, for example, by chromatography or spectrophotometry. Thus, the permeation kinetic profile and the amount of retained drug in the mucosa can be measured. Considering the local application, it is advantageous for the drug to slowly permeate the mucosa without reaching blood vessels and systemic circulation [8].

The cumulative permeation percentage was calculated after each time point in porcine oral mucosa. However, even after 24 hours no drug in the receptor compartment was detected suggesting that the amount of CUR was below the detection limit for the chromatographic method used.

The retention of CUR in porcine oral mucosa was 6.99 ± 0.49% (or 47.67 ± 3.33 µg/cm^3^), which demonstrates that the drug was retained in the mucosa but did not reach the blood flow. These results were influenced by the absence of the water in the donor acceptor. This condition promoted the relation with permeation by the PAS technique. These results were favorable for local application over the initial stages (Stage 0 – carcinoma in situ, Stage I – less than 2 cm tumor and Stage II – more than 2 and less than 4 cm tumor) after surgical procedure. Additionally, localized and initial tumors enable the choice of less aggressive treatments, such as the administration of nanostructured CUR [26–27]. According to Sannomiya and Furukawa [84], the surgical procedure is indicated for buccal squamous cells in initial stages, achieving tissue without tumor margin. For more advanced tumors, the use of chemotherapy and radiotherapy adjuvants is recommended [84].

**Figure 11:**
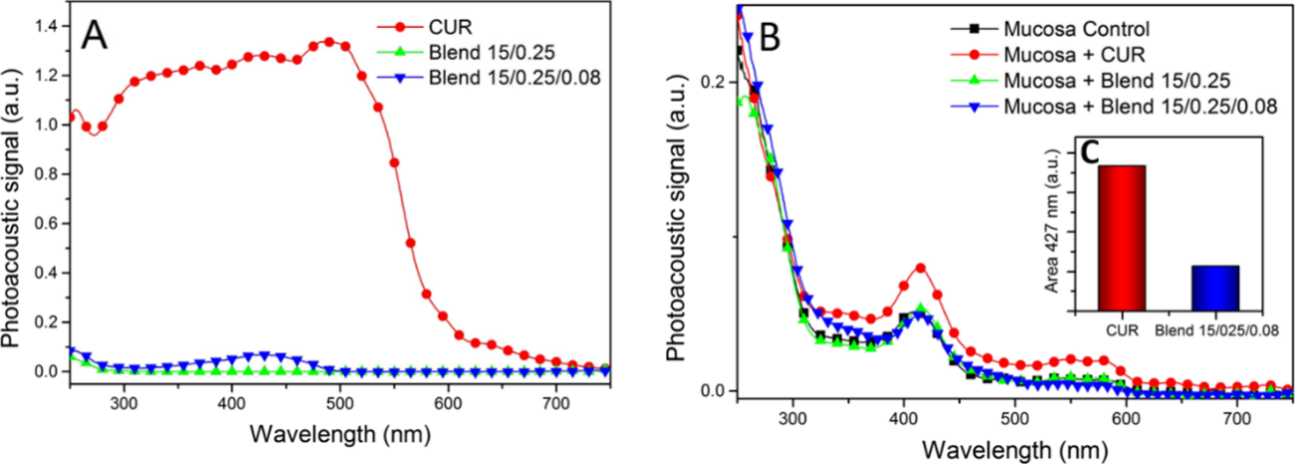
Optical absorption spectra obtained from PAS with (A) CUR and polymeric systems with and without CUR, (B) permeation of CUR through mucosa and (C) area of behavior of permeation of absorption band in 427 nm in mucosa.

Photoacoustic spectroscopy is useful in the investigation of permeation and distribution of substances in biological tissues in vitro, ex vivo and in vivo. This technique is based on the determination of optical spectral absorption by a photoacoustic signal created by the interaction of matter with radiation of a known wavelength [7,85]. Besides, the relatively low cost, non-destructible manner, and ability to detect low amounts of sample are interesting for investigation of opaque samples [7]. Moreover, this is a qualitative technique that determines if a drug can permeate or not and, if so, the depth of tissue that can be permeated. The permeation of CUR from polymer blends containing P407 and C974P^®^ were performed in porcine mucosa by photoacoustic spectroscopy and the photoacoustic spectra of the formulations, tissues and permeation of CUR are shown in Figure 11.

The optical absorption spectra of CUR and formulations are presented in Figure 11A. A Gaussian adjustment was performed in order to decompose the spectra into its components. It can be observed that the drug exhibits an intense band that varies from 250 to 700 nm, with peaks around 250, 315, 427, 515 and 659 nm. The main absorption band of CUR is located at 427 nm and is due to the aromatic rings of hydroxyl groups and ether [86]. The polymer blend 15/0.25 demonstrated a large band that varies from 250 to 350 nm, with peaks at 250 to 295 nm. On the other hand, the blend 15/0.25/0.08 displays variation from 250 to 500 nm with peaks at 250, 295, 315, 359 and 427 nm. In this sense, the detection of these characteristic bands in the mucosa is indicative of the presence of the drug.

Regarding the photoacoustic spectra of porcine oral mucosa (Figure 11B), all samples displayed a band at 415 nm related to the blood vessels. Figure 9C exhibits that CUR from formulations could permeate the mucosa. Moreover, the thermal diffusion length (µ_s_) was 31 µm on both sides of the oral mucosa. Thus, CUR permeated the total sample thickness (818 µm).

In this sense, the results of the PAS and Franz cell technique are complementary, since PAS can elucidate if the drug could permeate and the former is able to quantify the concentration of drug that went through the receptor vessel and was retained in the mucosa.

#### Drug and formulation cytotoxicity

**Figure 12:**
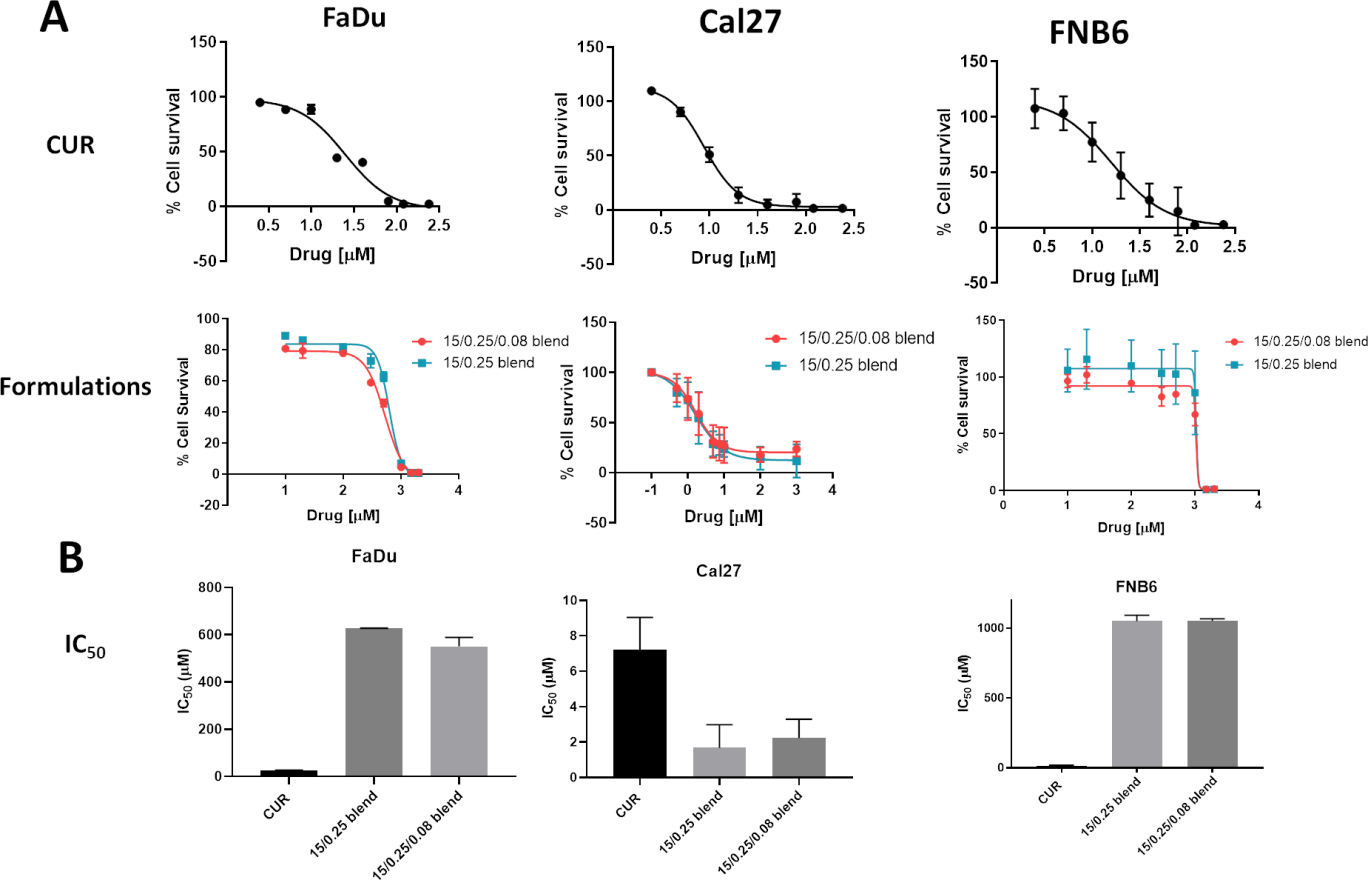
Cytotoxicity of formulations: (A) cell survival curves of drug and formulations, (B) IC_50_ comparisons of drug and formulations for two SCC (FaDu and Cal27) and one normal oral (FNB6) cell lines. Each IC_50_ value and percent cell survival represent average ± SEM (*n* = 3). CUR dispersed in DMSO aqueous solution exhibited cytotoxicity on all three cell types with average IC_50_ of 24.90 ± 2.30, 7.205 ± 1.831, 15.916 ± 3.440 µM, for FaDu, Cal27 and FNB6, respectively. The possible DMSO interference on methodology was investigated (Supporting Information File 1, Figure S2).

The cytotoxicity potential of the drug and formulations with and without CUR were investigated on squamous carcinoma cells (FaDu and Cal27) and normal oral immortalized keratinocytes (FNB6). The three cell lines were exposed to a wide range of drug concentrations (0, 2.5, 5, 10, 20, 40, 80, 120 and 240 µM) for 24 h. Moreover, another range of drug concentrations (2000, 1500, 1000, 500, 300, 100, 20, 10 µM) was utilized for the formulations. After this period, cell viability was indirectly determined by MTT metabolic assay (Figure 12).

The viability of Cal27 was significantly decreased (*p* < 0.05) by the presence of the formulations, regardless of the presence of CUR. This cytotoxic effect was not observed in the FaDu and FNB6 cells. The cytotoxic effect of the formulations could be explained by the presence of P407, a known surfactant. The incorporation of CUR into nanostructured systems caused a significant (*p* < 0.05) increase in the IC_50_ values for both FaDu and FNB6 compared to Cal27.

This behavior indicated that CUR could be released and permeate before it could kill the cells. Moreover, the presence of CUR significantly decreased the IC_50_ due to its cytotoxicity properties [87]. The formulations were diluted in order to maintain the viability of the cells. Therefore, CUR is released into the medium that has a large amount of organic molecules and low water activity to disperse the drug. However, each cell line was cultivated in a different type of medium, DMEM, RPMI and Green’s medium for Cal27, FaDu and FNB6, respectively. These different media could explain the results. The Cal27 viability was quite similar when treated with the drug and with the formulations, thus the same drug concentration is likely available. The slow drug release, ex vivo retention of CUR in the mucosa and the cytotoxic results suggest that these formulations would not be effective in primary therapy. However, it could be very useful after surgical procedure in order to kill the remaining cells.

## Conclusion

The physicochemical, mechanical, pharmaceutical and biological properties of mucoadhesive nanostructured systems containing CUR were explored in this study. The photophysical interactions, CUR incorporation kinetics and the location of drug in the micelles were elucidated. The pharmaceutical aspects, including rheology, mechanical properties, CUR release and permeation using two complementary methods were also investigated. Moreover, the biological characterization involved investigation of the cytotoxicity in tumor and healthy cell lines. The formulations prepared from solid dispersions stored at 25 °C containing P407, C974P and CUR displayed viscoelastic properties, plastic behavior with rheopexy at body temperature (which provides better retention), and increased mucoadhesive force due to the presence of CUR. CUR tends to be located in the core of the micelles; consequently, the drug displayed slow and complete release after 8 h but did not permeate the porcine oral mucosa. The cytotoxicity studies revealed the increase in the cytotoxic effects for some tumor cell lines (Cal 27) when incorporated into the formulation but decreased cytotoxic effects in healthy cells. Therefore, the nanostructured system demonstrated promising results due to the selectivity towards cancer cells in a monolayer cell culture in addition to exhibiting excellent physicochemical properties. Hence, further activity studies should be performed in tissue-engineered and in vivo models in order to test the performance of these systems in a more complex environment.

## Experimental

### Materials

Curcumin (>98% purity), poloxamer 407 and mucin from porcine stomach type II were purchased from Sigma-Aldrich (St. Louis, MO, USA). Carbopol 974P^®^ was kindly donated by Lubrizol (São Paulo, SP, Brazil). Curcumin C3 complex^®^ was received from Sabinsa^®^ (West Windsor, USA) and triethanolamine, used as a neutralizing agent, was purchased from Galena (Campinas, SP, Brazil). Potassium iodide was purchased from Biotec (São Paulo, SP, Brazil), sodium chloride was purchased from Nuclear (Diadema, SP, Brazil) and polyssorbate 80 (Tween 80^®^) came from Synth^®^ (Diadema, SP, Brazil).

Dulbecco’s Modified Eagle’s Medium high glucose (DMEM), fetal bovine serum (FBS), ʟ-glutamine, penicillin, streptomycin and trypsin were purchased from Sigma-Aldrich (St. Louis, MO, USA).

### Preparation of formulations

C974P (0.25%, w/w) was dispersed in purified water using a mechanical stirrer until complete dispersion. P407 (15%, w/w) was added to this mixture and the preparation was stored at 4 °C to ensure the complete wetting of the compounds. After 12 h, the formulations were stirred to provide the complete mixture of the two polymers. The final preparation was neutralized with triethanolamine, centrifuged at 3000 rpm to remove air bubbles, and stored at 4 °C for at least 24 h before further analysis [1,10–11]. Regarding the nanostructured systems containing curcumin, 15% (w/w) P407 was dispersed in ethanol and 0.08% (w/w) curcumin was added to the mixture and homogenized until complete mixture. The ethanol was eliminated using a rotary evaporator at 60 °C. When a thin film was obtained, the preparation was stored in a desiccator for 24 h. Afterwards, the thin film was added to the dispersion containing a mucoadhesive polymer (C974P), which was previously prepared. The system was stirred and finally the pH was adjusted to 7.0 using triethanolamine.

The nanostructured system containing CUR was also prepared by a second method (direct addition of CUR after preparation of binary polymeric system). Firstly, 0.25% of C974P was added to purified water and agitated until complete dispersion. Subsequently, 15% P407 was added to this mixture and stored in the refrigerator for 12 h. Afterwards, the mixture was agitated, CUR (0.08%, w/w) was added and the system pH was adjusted to 7 with triethanolamine.

### Interaction studies of curcumin in mucoadhesive nanostructured systems

#### Interaction evaluation by photophysical studies

The interaction between CUR and the polymer blend was evaluated by fluorescence spectrophotometry, where the formulations were diluted in water and the final concentration of the components were 1.8 × 10^−5^ mol/L curcumin, 0.01% (w/w) P407 and 0.0001675% (w/w) C974P. The behavior of the formulations was monitored regarding the increase of temperature and pH changes in the fluorescence emission spectra and anisotropy values obtained by the fluorescence spectrophotometer (Varian Agilent Technologies^®^). The anisotropy (*r*) was automatically calculated by the software Eclipse ADL Program Selector, according to the Equation 3:

[3] $$r=\frac{I_{\text{VV}}-GI_{\text{VH}}}{I_{\text{VV}}+2GI_{\text{VH}}}$$

where *I*_VH_ and *I*_VV_ represent the intensity measured by the excitation of the vertically aligned polarizer and the horizontally aligned polarizer, respectively. *G* is the instrumental correction factor of the ratio of the sensitivities for vertically and horizontally polarized light [48,88–89].

The excitation wavelength was 422 nm with emission wavelength 440 to 700 nm and the emission slit was set to 5–10 nm. All the systems were evaluated at 10 (below the critical micellar temperature), 25, 37 and 45 °C (above the critical micellar temperature) at pH 7. In addition, the systems containing P407 and CUR were evaluated at pH 7 and 10 at a temperature of 37 °C. At the same time, the binary polymeric systems were monitored in pH 5, 7 and 10 at 37 °C. All the measurements were performed after thermal equilibrium was achieved [46,90–91].

#### Studies of curcumin incorporation kinetics

In order to simulate the mechanism of incorporation of curcumin and the required time for the drug to reach the core of the polymeric micelles, the incorporation kinetic profile was determined using the CUR addition sequence based on the second method of preparation. In a quartz cuvette, 22 µL of CUR stock solution (4.8 × 10^−3^ mol/L) was added to the P407 and C974P polymeric dispersion without pH adjustment, totaling 3 mL. The final concentration was 3.6 × 10^−5^ mol/L CUR, 0.02% (w/w) P407 and 0.0032% (w/w) C974P. The kinetic profile was evaluated at 25 °C and 37 °C, over 125 min by monitoring the fluorescence emission spectra, where the excitation wavelength was 422 nm and the emission slit was set to 5–10 nm [92].

#### Localization of curcumin in nanostructured systems

The relative location of curcumin in the polymer blends containing P407, C974P and CUR prepared by solid dispersion stored at 25 °C and 5 °C was performed using iodide (I^−^) as a hydrophilic suppressor. Firstly, 133 µL of gel containing CUR and purified water were added to a 100 mL volumetric flask. Subsequently, increasing aliquots of KI (1 mol/L) were added in a solution containing polymeric micelles of CUR (3.6 × 10^−5^ mol/L), 0.02% (w/w) P407, and 0.0032% (w/w) C974P. The spectral emission profile from 440 to 700 nm was monitored after each iodide addition using an excitation wavelength of 422 nm and in most cases the emission slit was set to 5–10 nm. The Stern–Volmer (*K*_sv_) constant was obtained at 25 and 37 °C by Equation 4:

[4] $$\frac{F_{0}}{F}=1+K_{\text{sv}}\cdot \left[I^{-}\right]$$

where the emission values in the presence and absence of a suppressor are represented by *F*_0_ and *F*, respectively, and the concentration of iodide in the solution is given by [*I^−^*] [91,93–94]. The dilution effect promoted by the addition of each hydrophilic suppressor aliquot was corrected for each spectral emission profile.

#### Morphological analysis by scanning electron microscopy

The morphological characteristics of formulations in the presence and absence of curcumin were evaluated by an electron scanning microscope (Quanta FEI, Thermo^®^, Oregon, USA). Approximately 2 g of the formulation was freeze-dried and a sample of material was placed on double-sided tape, and the sample was coated with colloidal gold under argon atmosphere.

#### Morphological analysis by transmittance electron microscopy

The morphology of the formulations was also determined using a JOEL JEM 1400 transmission electron microscope (Peabody, MA, USA). 0.2 mL of the nanostructured material was diluted in a 10 mL volumetric flask and placed on a formvar/Carbon 200 mesh, copper grid (Ted Tella, Redding, CA, USA). This set was negatively stained with 2% (w/v) uranyl acetate solution for observation [57,95–96]. The samples were prepared at 37 °C.

#### Micelle size analysis

The hydrodynamic diameter (*D*), polydispersity index (PDI) and size distribution of 10%, 50% and 90% (D10%, D50% and D90%) of micelles of the nanostructured systems was carried out by dynamic light scattering (DLS) analysis using a NanoPlus Particle Size Analyzer (Particulate Systems, Norcross, GA, USA). The formulation samples were diluted 50 and 10 times to provide P407 concentrations of 0.3% and 1.5% (w/w), respectively. The measurements were performed at 25 °C and 37 °C with at least three replicates.

#### Rheometry

**Figure 13:**
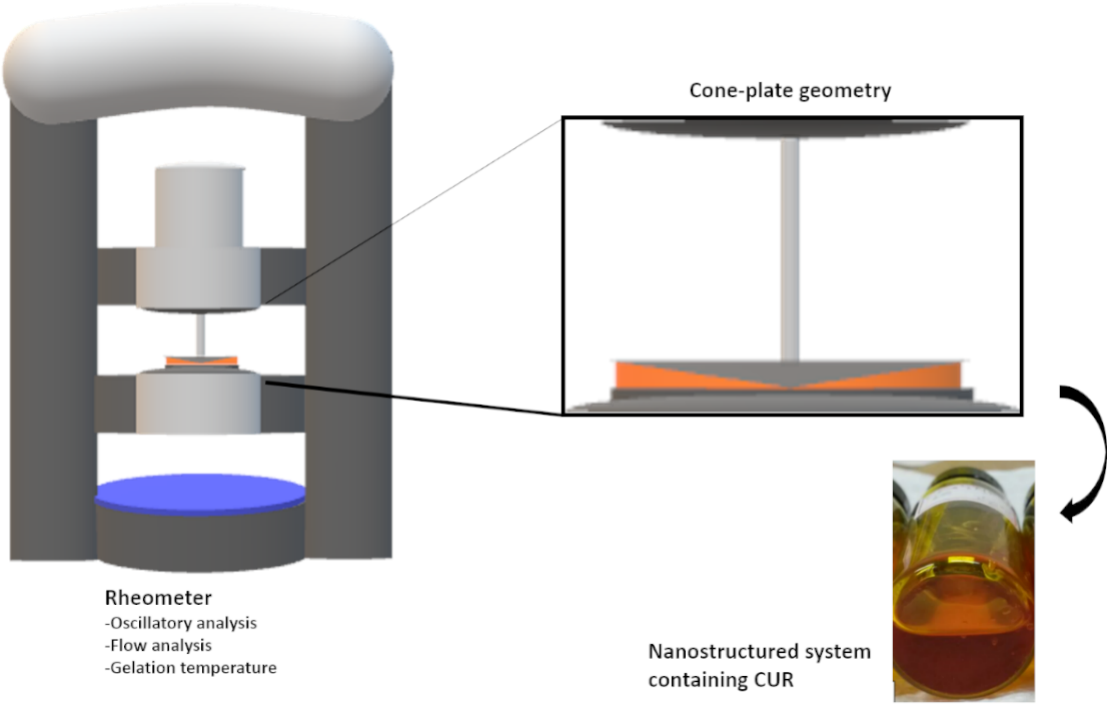
Schematic presentation of the rheological analysis setup utilized for continuous shear (flow) and oscillatory rheometry analysis of the formulations.

The rheological analysis of the formulations was determined using a controlled stress rheometer (MARSII, Haake Thermo Fisher Scientific Inc., Newington, Germany) at 25 °C and 37 ± 0.1 °C with a geometry employing a parallel steel cone-plate (35 mm diameter, separated by a fixed distance of 0.052 mm, where the cone angle is 2°), as shown in Figure 13.

The samples were carefully placed in the device, and it was allowed to equilibrate for at least 1 min before analysis to ensure the minimized shearing of the sample.

#### Continuous shear flow rheology

In flow mode, the downward and upward curves were obtained over shear rates from 0 to 2000 s^−1^, increasing over a period of 150 s, retained at the high limit during 10 s, and then decreasing over a period of 150 s. The flow properties were determined from at least five replicates and the upward flow curves were modelled using the power-law fluid (or the Ostwald–de Waele) relationship (Equation 1) [28,31,47]:

[1]
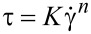


where τ is shear stress (Pa), *K* is the consistency index [(Pa·s)*^n^*], γ̇ is shear rate (s^−1^), and *n* is the flow behavior index (dimensionless).

The yield stress was evaluated by the rheological models of Casson (Equation 5) and Herschel–Bulkley (Equation 6) [97]:

[5]
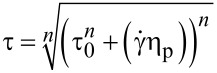


[6]
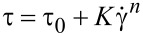


where τ_o_ is yield stress (Pa) and η_p_ is the Casson plastic viscosity. Moreover, the hysteresis area was calculated by the software RheoWin 4.10.0000 (Haake^®^).

#### Oscillatory rheology

In oscillatory mode, the linear viscoelastic region (LVR) was determined for each binary polymeric system. Subsequently, the frequency sweep analysis was performed from 0.1 to 10.0 Hz at 25 and 37 °C. The viscoelastic properties of the preparations, storage modulus (G’), loss modulus (G”), dynamic viscosity (η’) and the loss tangent (tan δ) were calculated using the software RheoWin 4.10.0000 (Haake^®^). The analyses were performed at least in five replicate samples [27–28,98–99].

#### Sol–gel transition temperature

In oscillatory mode with a controlled temperature ramp, the sol–gel transition temperature (*T*_sol–gel_) of the formulations was investigated as well. The LVR of each system was performed at 5 °C and 60 °C. Afterwards, over a range of 5–60 °C, the temperature sweep analysis was performed at a defined frequency (1.0 Hz) with a heating rate of 10 °C/min with controlled stress. The viscoelastic properties, G’, G”, η’ and tan δ were calculated using the software RheoWin 4.10.0000 (Haake^®^) with at least five replicate samples in each case. *T*_sol–gel_ is defined as the temperature at which G’ was halfway between the values for solution and gel and was calculated for all preparations where η’ increased with the significant increase of temperature [47,49–51].

#### Texture profile analysis

The texture profile analysis (TPA) of preparations with and without CUR was carried out using a texture profile analyzer TA-XTplus (Stable Micro Systems^®^, Surrey, UK) at 25 and 37 °C, in TPA mode, for at least three replicates [59]. A 13 g formulation was compressed at a depth of 15 mm, two times, by an analytical probe of polycarbonate (10 mm diameter) at a speed of 2 mm/s and with 15 s between the first and the beginning of the second compression. The resultant force versus distance plot provided the texture parameters, hardness, compressibility, adhesiveness, elasticity and cohesiveness [59].

### Mucoadhesive properties

#### In vitro evaluation of mucoadhesive strength by detachment force

The mucoadhesive properties of the formulations (with and without CUR) were investigated using a texture analyser (TA-XTplus, Stable Micro Systems^®^) in tension mode at 37 °C, repeated at least three times. Firstly, the mucin disc was prepared by the compression of crude mucin, hydrated in mucin solution 5% (w/w) for 30 s and fixed in the TPA probe. The excess liquid was gently removed with absorbent paper. The samples were placed behind the analytical probe, which was lowered until both surfaces were in contact. Subsequently, a force of 0.1 N was applied for 30 s in order to ensure the intimate contact between the mucin disc and sample. Afterwards, the probe was raised at speed of 1.0 mm/s and the force required to detach the mucin disc from the formulations was determined from the plot of force versus time.

#### Ex vivo analysis of mucoadhesive properties by falling liquid method

The mucoadhesive properties of the formulations were also investigated by an ex vivo methodology using porcine oral mucosa on a flow through method. Porcine oral mucosa was taken from the cheek of young, white and freshly slaughtered pigs from a slaughterhouse authorized for human consumption by the Brazilian Agriculture Ministry. After the oral mucosa was excised with scissors and a surgical scalpel, the samples were stored at −18 °C in a PBS solution and defrosted at room temperature on the experimental day (48 hours after the oral mucosa preparation) [30].

**Figure 14:**
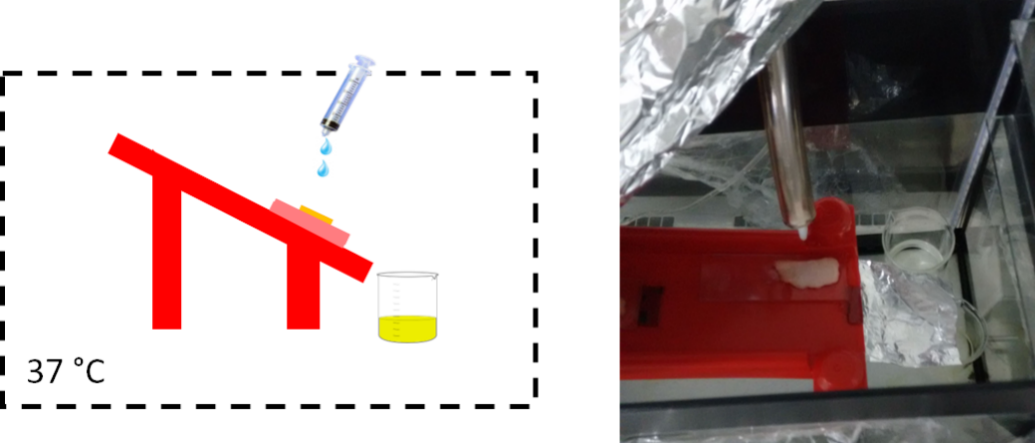
Schematic presentation of the setup utilized for ex vivo liquid falling mucoadhesion tests.

Inside a temperature-controlled chamber (37 °C), the oral mucosa samples were placed on the test channel behind a syringe-pump system, where phosphate sodium buffer was dropped over the mucosal samples (flow of 4 mL/min) (Figure 14). 100 µL of the formulation was placed over the oral mucosa and kept warm, in order to allow the adhesion between the mucosa and formulation. After 5 min, PBS was flowed over the set for 20 min. The samples of the elution liquid were collected after 1, 2, 5, 10, 15 and 20 min, with 1% (v/v) Tween 80 to allow the complete dispersion of CUR. The samples were diluted with methanol (1:2) and analyzed by the HPLC method. 20 µL of the sample was injected into a Shimadzu LC CBM 20 system (Tokyo, Japan) equipped with a UV–vis detector (SPD 20 A) and manual injector (7725i). A C18 reversed phase column (5 µm × 4.6 mm × 250 mm, Luna PFP, Phenomenex^®^, Torrance, USA) was used as a stationary phase and the mobile phase was acetonitrile and acetic acid solution (1.5%, v/v) in a gradient elution. The flow was adjusted to 1.0 mL/min and the peak area was detected at 425 nm. The amount of formulation removed from the surface of the substrate was calculated and deducted from the total, providing the retention data. The analysis was performed in triplicate using new oral mucosa for each experimental essay [10,52–53].

#### Syringeability determination

The formulation resistance to compression inside a syringe was determined by the syringeability work. The investigations were performed in a texture analyzer (TA-XTplus) in compression mode [100]. In order to avoid the entrapment of air, the formulations were carefully packed in 1 mL plastic syringes at 30 mm. Each syringe was vertically fixed in the texture meter and pressed at 2.0 mm·s^−1^, to a depth of 30 mm, until initial contact with the syringe plunger was made. The analysis was performed at 25 °C with at least three replicates [47,55]. During the compression of the plunger, a graph of force versus distance was derived and the work demonstrates the resistance of the compression of syringe content.

#### In vitro drug release

**Figure 15:**
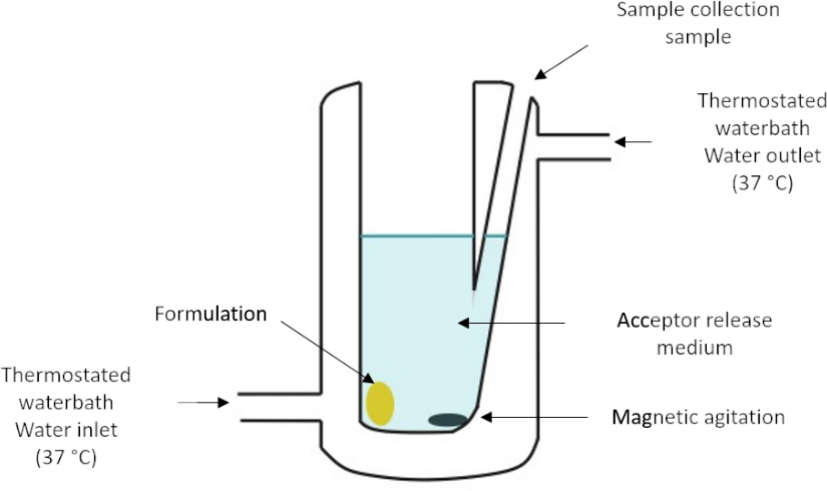
Schematic presentation of the modified Franz’s cell used for the in vitro curcumin release measurements from the nanostructured system.

The kinetics of the release of curcumin from the binary polymeric system was carried out using double-wall glass beaker with water bath temperature control at 37 ± 0.5 °C. 1.0 g polymeric system was placed on the bottom of the vessel at 37 °C to ensure the complete gelation of the formulation. Subsequently, 16 mL of the release media (Tween 80 aqueous solution; 1%, v/v) was carefully added in the recipient to obtain sink conditions and kept under constant agitation (Figure 15) [11,56,101]. 500 µL aliquots of samples were collected and replaced with the same volume of fresh medium at fixed time intervals, 0.5 h, 1 h, 2 h, 3 h, 4 h, 6 h, 8 h and 24 h. These samples were diluted with methanol (1:2), filtered with PTFE membranes and quantified by the HPLC method, as previously described [30]. The release profiles were calculated by plotting the amount released versus time. These release profiles were fitted with the Korsmeyer–Peppas equation (Equation 7), which describes the drug release from matrix polymeric systems [102]:

[7] $$F=k\cdot t^{n}$$

where *F* represents the fraction of the drug released, *t* is the time released, *k* is the combined kinetic constant of structural and geometric characteristics of the apparatus and *n* is the release exponent, which reveals the drug release mechanism.

### Ex vivo analysis of curcumin permeation in oral mucosa

#### Mucosa permeation by Franz cells

The permeation was investigated using porcine oral mucosa obtained as previously described in the section on ex vivo analysis of mucoadhesive properties by falling liquid method. Firstly, the tissues were defrosted at room temperature, then cut and placed between the donor and receptor compartment of the Franz cell. The receptor media, composed of 1% (v/v) Tween 80 in PBS buffer (pH 7.4), which provided the solublization of CUR, was placed in the receptor compartment. This system was maintained at 37 °C at constant stirring. 1 mL of the formulation was placed homogeneously over the mucosa in the donor compartment. The temperature of the formulation was maintained at 37 °C by thermal exchange between the tissue and the formulation and also the temperature control provided by the equipment [103–104]. Afterwards, 500 µL of the receptor medium was collected and replaced by fresh medium to ensure sink condition at 0.5 h, 1 h, 2 h, 3 h, 4 h, 6 h, 8 h and 24 h. The samples were diluted in methanol (1:2), filtered in PTFE and quantified by the HPLC method. At least three replicates were performed [56,61].

For the retention of CUR in the tissue, the porcine mucosa was taken from the recipient. The tissue was cut into small pieces and placed in a 5 mL volumetric flask with methanol and sonicated for 15 min. Subsequently, the 5 mL volumetric flask was completed, filtered with PTFE and quantified using the HPLC method. The analysis was performed for at least three samples.

#### Mucosal permeation by photoacoustic spectroscopy

The permeation of curcumin from the mucoadhesive thermoresponsive systems, performed in porcine oral mucosa, was investigated by photoacoustic spectroscopy. The oral mucosa was taken from porcine cheek as described previously. Firstly, 30 µg of the formulation was homogeneously placed over a 1 cm^3^ surface of the oral mucosa. After 30 min, the sample was evaluated by photoacoustic spectroscopy. This essay was performed on home-built experimental equipment composed of a 1000 W Xenon arc lamp (Oriel, model 68820) as the light source with a nominal power of 800 W. The light was diffracted when passing through the 3.16 mm input and output slits of the monochromator (Oriel, model 77250) and then modulated at 13 Hz with a mechanical chopper (Stanford Research Systems, model SR 540) and then focused on the sample. Band-pass filters were used to eliminate higher order diffraction. The sample was placed inside the photoacoustic cell and sealed with a transparent quartz window (diameter of 8 mm and thickness of 2 mm). The photoacoustic signal generated by pressure changes resulting from the periodic heating of the sample was collected by a capacitive microphone (Brüel and Klaer, model 2669). A lock-in amplifier by EG&G Instruments, model 5110, was used. The thermal diffusion length (µs) was used to calculate the depth of tissue that contributed to the photoacoustic signal:

[2] $$\mu_{\text{s}}=\sqrt{\frac{D}{\mu f}}$$

where *D* is the sample thermal diffusivity (cm^−2^·s^−1^) and *f* is the light modulation frequency (Hz). Considering the thermal diffusivity of mucosa equal to skin, due to the similarity overall in terms of molecule distribution and histological architecture [105], *D* = 4.1 × 10^−4^ cm^2^·s^−1^ [106–107] and *f* =13 Hz. Consequently, µ_s_ was 31 µm for oral mucosa and the total sample thickness was constant around 818 µm.

The photoacoustic signal was interpreted from the band absorption spectra, since the final photoacoustic signal is proportional to the sample absorption coefficient [107]. All spectra were normalized with a sample of carbon black in order to correct the source emission intensity in each wavelength [108].

Moreover, the spectra of at least three porcine oral mucosa were obtained by placing the tissue inside the photoacoustic cell and illuminating the side to be measured; then the tissue was turned upside down to illuminate the opposite side [56,60].

#### Cell culture

The cell lines FaDu (LGC Promochem, Middlesex, UK), originally isolated from a hypopharyngeal tumor and Cal27 (ATCC, Manassas, VA, USA, CRL-2095) from tongue squamous cell carcinoma (ECACC, Health Protection Agency Culture Collections, Salisbury, UK) were used in this study. FaDu cells were cultured in RPMI-1640, whereas Cal27 was cultured in Dulbecco’s modified Eagle’s (DMEM) medium high glucose. Both media were supplemented with 10% (v/v) fetal bovine serum (FBS), 2 mM ʟ-glutamine, 100 UI/mL penicillin and 100 µg/mL streptomycin. The immortalized cell line FNB6 (a kind gift from Professor Keith Hunter), originally isolated from normal oral keratinocytes, were cultured in adenine enriched medium; DMEM and Ham’s F12 medium in a 3:1 (v/v) supplemented with 10% (v/v) FBS, 0.1 mM cholera toxin, 10 ng/mL epidermal growing factor, 0.4 µg/mL hydrocortisone, 0.18 mM adenine, 5 mg/mL transferrin, 2 mM ʟ-glutamine, 0.2 mM triiodothyronine, 0.625 mg/mL amphotericin B, 100 UI/mL penicillin and 100 µg/mL streptomycin. All cells were incubated at 37 °C in a 5% CO_2_ humidified atmosphere and sub-cultivated using trypsin-EDTA when 80% confluence was reached.

#### Cytotoxicity and biological activity evaluation

The in vitro cytotoxicity of preparations with and without CUR, as well as the drug alone, were carried out on FNB6, Cal27 and FaDu cells using an MTT assay as previously described [85]. Briefly, 2 × 10^5^ cells were seeded in each well of a 96-well plate before addition of the formulations with and without CUR with increased polymeric content (2000, 1500, 1000, 500, 300, 100, 20, 10 µM CUR) and free CUR (240,120, 80, 40, 20, 10, 5 and 2.5 µM). After 24 h, the media with CUR and/or the formulations was aspirated, the cells were washed three times with PBS and more 200 µL media was added to each well and the cells were incubated for a further 24 h. Monolayer cultures were incubated for 1 h at 37 °C with 0.5 mg/mL MTT solution, after which the solution removed and acidified isopropanol was added to remove the blue formazan crystals. The optical density was measured at 570 nm with a 630 nm reference correction.

#### Statistical analysis

The effect of CUR presence and temperature in micelle size analysis, consistency index (*K*), flow index (*n*), yield value, hysteresis area and texture profile analysis parameters (hardness, compressibility, adhesiveness, elasticity and cohesiveness) were statistically compared using two-way ANOVA. On the other hand, the effect of the presence of CUR on the mucoadhesive strength, softness and syringeability were statistically evaluated by one-way ANOVA. Moreover, the effect of the drug and formulation on cell viability was evaluated by two-way ANOVA. Student *t*-test was used to determine the influence of temperature on dynamic viscosity of the formulations. All the cases of ANOVA post hoc comparison of individual groups were carried out using Tukey. For all cases, *p* < 0.05 was accepted as significant [23,28,31].

## Supporting Information

File 1 The results of the evaluation of the storage temperature of the formulations and the investigation on the DMSO interference in the CUR cytotoxicity studies.
