# Supplementary material for: Design of a nanostructured mucoadhesive system containing curcumin for buccal application: from physicochemical to biological aspects
Source: Beilstein J Nanotechnol. 2019 Nov 25;10:2304–28. doi: 10.3762/bjnano.10.222 (PMC6902884; doi:10.3762/bjnano.10.222)
Supplement: File 1 — The results of the evaluation of the storage temperature of the formulations and the investigation on the DMSO interference in the CUR cytotoxicity studies. [file Beilstein_J_Nanotechnol-10-2304-s001.pdf]

BEILSTEIN JOURNAL OF NANOTECHNOLOGY

Supporting Information

for

Design of a nanostructured mucoadhesive system containing curcumin for buccal application: from physicochemical to biological aspects

Sabrina Barbosa de Souza Ferreira, Gustavo Braga, Évelin Lemos Oliveira, Jéssica Bassi da Silva, Hélen Cássia Rosseto, Lidiane Vizioli de Castro Hoshino, Mauro Luciano Baesso, Wilker Caetano, Craig Murdoch, Helen Elizabeth Colley and Marcos Luciano Bruschi

Beilstein J. Nanotechnol. **2019,** *10,* 2304–2328. doi:10.3762/bjnano.10.222

The results of the evaluation of the storage temperature of the formulations and the investigation on the DMSO interference in the CUR cytotoxicity studies

License and Terms: This is a supporting information file under the terms of the Creative Commons Attribution License (http://creativecommons.org/licenses/by/4.0). Please note that the reuse, redistribution and reproduction in particular requires that the authors and source are credited.
The license is subject to the *Beilstein Journal of Nanotechnology* terms and conditions: (https://www.beilstein-journals.org/bjnano)

Evaluation of storage temperature of formulations

In order to confirm the best incorporation method described previously and storage temperature for CUR systems. The gels were prepared and 25 °C and their physical characteristics (appearance and phase separation) were evaluated after 15 days.

The formulations containing C974P before and after pH adjustment are shown in Figure S1.

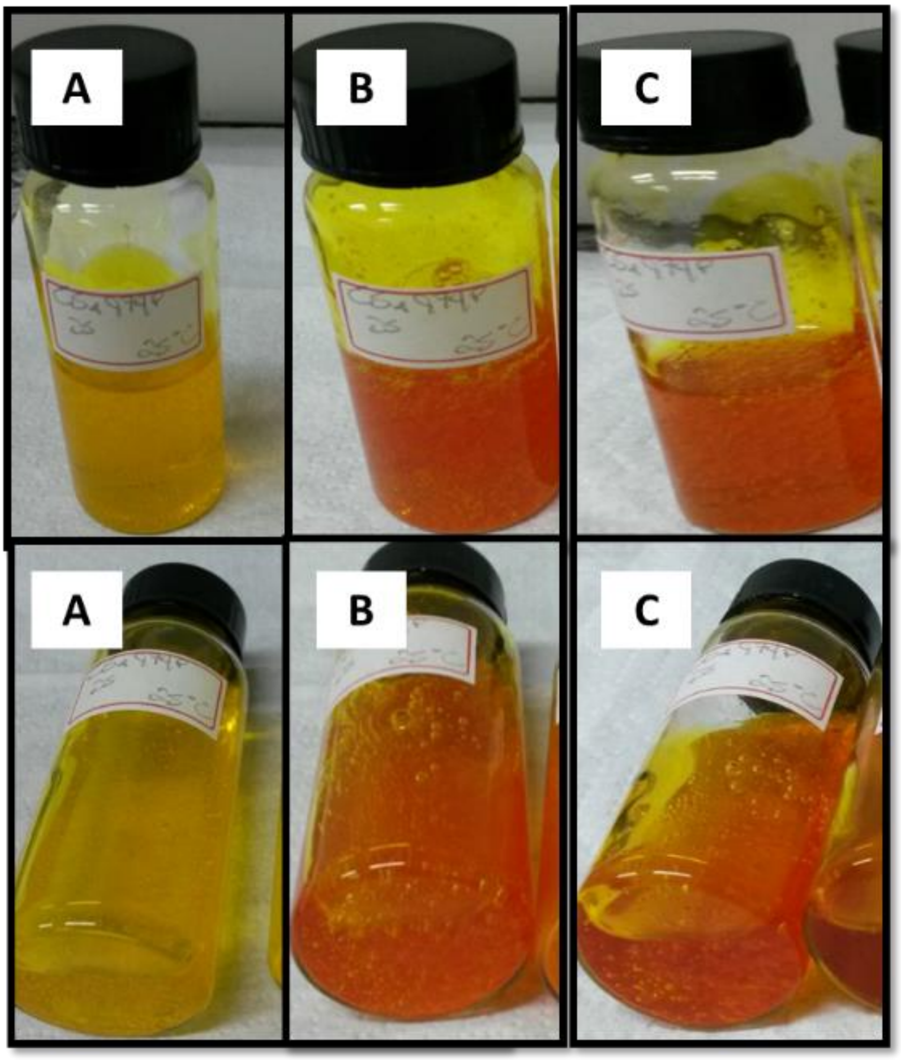

Figure S1: Binary polymeric systems containing 15% (w/w) P407, 0.25% (w/w) C974P and 0.1% CUR. Each photo represents the systems prepared by solid dispersion and stored at 25 °C, before (A) and after (B) pH adjustment and after 15 days of storage (C).

Alterations in the colour of the preparations before and after pH adjustment can be explained by the pK_a proximity of CUR (Figure S1) with some reports in the literature of CUR with pK_a values around 8, 9 and 10 [1]. After the pH adjustment, the formulations displayed higher viscosity and the presence of air bubbles, due to the cross-linking of C974P [2].

These preparations were stored for 15 days at 5 and 25 °C and, after this time, the systems did not display differences in their appearance and there was no evidence of CUR precipitation, which proves that the system is physically stable for 15 days (Figure S1).

Investigation about the DMSO interference in the CUR cytotoxicity studies

In order to eliminate the interference of DMSO in the cytotoxicity of CUR and enable the solubility of the drug, the cytotoxicity of DMSO against Cal27, FaDu and FNB6 was investigated. Initially, 2×10^5 cells were seed and cultivated until confluence in each well of a 96-well plate. After overnight culture, the increasing dilutions of DMSO in medium were added with final concentration of 0.1, 0.2, 0.5, 1, 2, 5, 10 and 20% (v/v) for 24 hours and along with a media only the negative control was media and cells. Subsequently, the media with DMSO was aspirated, cells were washed with buffer PBS (three times) and 200 µL media was added to each well. After a further 24 hours of incubation, 0.5 mg/mL MTT (Sigma, Poole Dorset, UK) solution was added to the cells, which were incubated for 1 hour at 37 °C. Finally, the solution was removed, and acidified isopropanol was added to solubilise blue formazan crystals. The absorbance was measured in a plate reader at 570 nm, with 620 nm reference correction.

CUR is a drug with strong hydrophobicity and light sensitive, as well as, susceptible to pH changes. In this sense, it is necessary to increase bioavailability by incorporating

compounds in the formulations to protect the drug, release the drug in a controlled-manner and at the same time does not interfere or decrease its biological activity [3]. Cytotoxicity tests were carried out with free drug, CUR-gel and gel without CUR, in order to evaluate the effect of the drug activity in cancerous and healthy cells, as well as, to investigate if the formulation without the drug killed the cells, hence representing a biocompatibility assay. To investigate the cytotoxicity of the free CUR, it was necessary to solubilise the drug in a solvent to increase its bioavailability yet at the same time would not interfere in the viability of the tested cell lines. Among them, dimethyl-sulfoxide (DMSO) [4-9] and ethanol 96% [10] have been reported to commonly solubilise CUR. Considering that the cell lines would be incubated with the drug for 24 hours in an incubator at 37 °C and the volatility of ethanol, this solvent would evaporate and consequently, the drug would precipitate and decrease the bioavailability of CUR to exert it biological activity; thus this solvent was not used to solubilise CUR. DMSO does not display volatility and would promote CUR solubility. Thus, it was the solvent chosen for the tests. However, this solvent has shown high toxicity for cells depending on the concentration of DMSO and metabolism of each cell line [11,12]. In general, 1% DMSO is used to solubilise hydrophobic drugs [13]. In this sense, the cytotoxicity was performed by the MTT assay in Cal27, FaDu and FNB6, where DMSO was diluted in the media (0.1, 0.25, 0.5, 1, 2, 5, 10 and 20%, v/v). In order to elucidate the DMSO concentrations that could solubilise CUR yet would not reduce the viability of the cells. Dose-response graphs of to determine the IC_{50} are displayed in Figure S2.

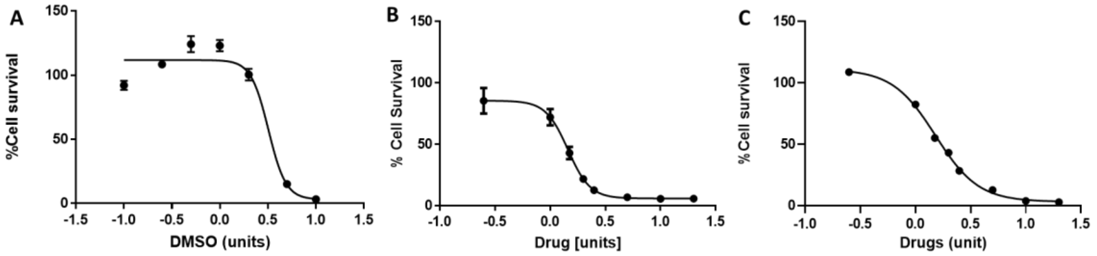

Figure S2: IC_{50} graphs of DMSO concentrations in cell lines: FaDu (A), Cal27 (B) and FNB6 (C).

FaDu cells were most resistant to the range of DMSO concentrations tested, where concentrations higher than 2% displayed a decrease in the cell viability with an IC_{50} of 3.2%. On the other hand, the other cell lines were more susceptible to the solvent with IC_{50} of 1.45% and 1.51% for Cal27 and FNB6, respectively, and were 100% viable in concentrations around 0.25% (V/V) DMSO. Considering that the higher concentration of CUR tested in the cells was 240 µM, the concentration of DMSO tested was 0.00624% (v/v) and, therefore, this solvent did not affect the viability of the cells.

References

1. Tonnesen, H.H.; Másson, M.;Loftsson, T. *Int. J. Pharm.* **2002**, 244, 127-135. doi:10.1016/S0378-5173(02)00323-X
2. De Souza Ferreira, S. B.; Da Silva, J. B.; Junqueira, M.V.; Borghi-Pangoni, F.B.; Gomes, R.G.; Bruschi, M.L.*J. Mech. Behav. Biomed. Mater.* **2017**, *74*, *142-153*. doi:10.1016/j.jmbbm.2017.05.040
3. de Souza Ferreira, S. B.; Bruschi, M. L. *Ther. Deliv.* **2019**, *10*, 83–86. doi:10.4155/tde-2018-0075
4. de Campos, P. S.; Matte, B. F.; Diel, L. F.; Jesus, L. H.; Bernardi, L.; Alves, A. M.; Rados, P. V.; Lamers, M. L. *Phyther. Res.* **2017**, *31*, 1433–1440.

doi:10.1002/ptr.5872

5. Dudás, J.; Fullár, A.; Romani, A.; Pritz, C.; Kovalszky, I.; Hans Schartinger, V.; Mathias Sprinzl, G.; Riechelmann, H. *Exp. Cell Res.* **2013**, *319* (6), 800–809. doi:10.1016/j.yexcr.2012.12.001
6. Kim, J. Y.; Cho, T. J.; Woo, B. H.; Choi, K. U.; Lee, C. H.; Ryu, M. H.; Park, H. R. *Arch. Oral Biol.* **2012**, *57*, 1018–1025. doi:10.1016/j.archoralbio.2012.04.005
7. Kunwar, A.; Barik, A.; Mishra, B.; Rathinasamy, K.; Pandey, R.; Priyadarsini, K. I. *Biochim. Biophys. Acta - Gen. Subj.* **2008**, *1780*, 673–679. doi:10.1016/j.bbagen.2007.11.016
8. Lee, M.-H.; Lin; Thomas; Chen; Shen; Yang; Yang, W.-J.; Thomas. *Int. J. Nanomedicine* **2012**, 7, 941-951. doi:10.2147/ijn.s28510
9. Liao, F.; Liu, L.; Luo, E.; Hu, J. *Arch. Oral Biol.* **2018**, *92*, 32–37. doi:10.1016/j.archoralbio.2018.04.015
10. Singh, R.; Tønnesen, H. H.; Kristensen, S.; Berg, K. *Photochem. Photobiol. Sci.* **2013**, *12*, 559–575. doi:10.1039/c2pp25249j
11. Hebling, J.; Bianchi, L.; Basso, F. G.; Scheffel, D. L.; Soares, D. G.; Carrilho, M. R. O.; Pashley, D. H.; Tjäderhane, L.; De Souza Costa, C. A. *Dent. Mater.* **2015**, *31*, 399–405. doi:10.1016/j.dental.2015.01.007
12. Pasley, S.; Zylberberg, C.; Matosevic, S. *Immunol. Lett.* **2017**, *192*, 35–41. doi:10.1016/j.imlet.2017.09.012
13. Adler, S.; Pellizzer, C.; Paparella, M.; Hartung, T.; Bremer, S. *Toxicol. Vitr.* **2006**, *20*, 265–271. doi:10.1016/j.tiv.2005.06.043
